# New Horizons in the Diagnosis of Gastric Cancer: The Importance of Selected Toll-like Receptors in Immunopathogenesis Depending on the Stage, Clinical Subtype, and Gender of Newly Diagnosed Patients

**DOI:** 10.3390/ijms25179264

**Published:** 2024-08-27

**Authors:** Marek Kos, Krzysztof Bojarski, Paulina Mertowska, Sebastian Mertowski, Piotr Tomaka, Łukasz Dziki, Ewelina Grywalska

**Affiliations:** 1 Department of Public Health, Medical University of Lublin, 1 Chodźki Street, 20-093 Lublin, Poland; marek.kos@umlub.pl; 2 General Surgery Department, SP ZOZ in Leczna, 52 Krasnystawska Street, 21-010 Leczna, Poland; k.bojarski@szpital.leczna.pl; 3 Department of Experimental Immunology, Medical University of Lublin, 4a Chodźki Street, 20-093 Lublin, Poland; sebastian.mertowski@umlub.pl (S.M.); ewelina.grywalska@umlub.pl (E.G.); 4 Department of Anesthesiology and Intensive Care, SP ZOZ in Leczna, 52 Krasnystawska Street, 21-010 Leczna, Poland; p.tomaka@szpital.leczna.pl; 5 Department of General and Oncological Surgery, Faculty of Medicine, Medical University of Lodz, 251 Street, 92-213 Lodz, Poland; lukasz.dziki@umed.lodz.pl

**Keywords:** gastric cancer Toll-like receptors, TLR2, TLR3, TLR4, TLR9, immune system

## Abstract

Introduction: Toll-like receptors (TLRs) play a vital role in the innate immune response, recognizing pathogens and initiating the inflammatory response. Research suggests that TLRs may also have a significant impact on the development and progression of cancers, including gastric cancer (GC). Understanding the role of individual TLRs in the immunopathogenesis of gastric cancer may provide new information necessary to develop more effective diagnostic and therapeutic methods. Aim of the study: This study aimed to determine the role of selected TLR-2, -3, -4, and -9 in the immunopathogenesis of patients with newly diagnosed and untreated gastric cancer. Materials and methods: The study included 60 newly diagnosed, untreated GC patients and 25 healthy volunteers. The research included analyses assessing the percentage of the tested TLRs on T and B lymphocyte subpopulations using multicolor flow cytometry and assessing their concentration in the serum of the examined patients using ELISA tests. The statistical analyses performed included a comparison of patients in individual stages of gastric cancer, an analysis of the most common clinical subtypes of gastric cancer, and a comparative analysis of differences in the gender of recruited patients. Results: Our studies showed different expression levels of TLR-2, -3, -4, and -9 on T and B lymphocyte subpopulations, as well as their different concentrations in patients’ serum. Significant differences in the expression of these receptors were observed depending on the stage of gastric cancer and its clinical subtypes. These differences were also visible in the context of patient gender. Summary: The results of our studies suggest that TLR-2, -3, -4, and -9 may play an important role in the immunopathogenesis of gastric cancer. The differential expression of these receptors depending on the stage of the disease, clinical subtype, and gender of patients may have potential diagnostic and therapeutic significance. Further research is necessary to understand better the mechanisms of action of TLRs in gastric cancer and to apply this knowledge in clinical practice.

## 1. Introduction

Gastric cancer (GC) is one of the most common and malignant forms of gastrointestinal cancer, contributing to high mortality worldwide. According to data for 2022 made available by the World Cancer Research Fund International (WCRF) and the International Agency for Research on Cancer WHO (IARC), as well as based on data from the available literature, it is the fifth most common cancer in the world, while in terms of gender, men are ranked in fourth place and women in seventh place [1,2,3,4]. This cancer shows significant geographical variation and is considered a global health problem. The highest incidence rates occur in East Asia, especially Japan, South Korea, and China. Mongolia, however, has the highest rate of both disease and death in the world. High incidence rates are also observed in Eastern Europe and Central and South America [2,5]. It develops in the gastric mucosa’s glandular cells, forming adenocarcinoma. This process occurs slowly, often over many years, which makes early detection difficult and, consequently, quick implementation of treatment, lowering the chances of cure. Initial symptoms such as indigestion, abdominal pain, or loss of appetite are subtle and non-specific, which often leads to misdiagnosis as other digestive problems. More pronounced symptoms, such as weight loss, difficulty swallowing, vomiting, or black stools, appear only in advanced stages [6,7,8].

In the oncological literature, there are two main histological types of GC: intestinal type and diffuse type, according to Lauren’s classification [9]. Both types differ in morphological and pathogenetic terms, which is essential for therapeutic strategies. Intestinal-type GC is characterized by well-differentiated tumor cells forming glandular structures resembling those found in the intestine, which results in a slightly better prognosis. This type of cancer is more common in older people and is more common in men than women. It develops gradually, often as a result of chronic gastritis caused by *Helicobacter pylori* infection, which is responsible for 75–90% of stomach cancer cases. Additionally, long-term exposure to risk factors, such as an incorrect diet (rich in salted, smoked, and pickled products) and genotoxic substances such as nitrates and nitrosamines resulting from, e.g., smoking, also play a significant role in the pathogenesis of this cancer. Because of the more defined tumor boundaries, resection with a smaller margin (approx. 3–5 cm) may be sufficient for this type of cancer. Surgical excision of the tumor with a margin of healthy tissue aims to remove all cancer cells with minimal damage to surrounding healthy tissue [10,11,12,13,14,15]. In turn, diffuse GC is characterized by poorly differentiated tumor cells diffusely infiltrating the gastric wall, leading to stiffness and thickening of the organ wall. It is more difficult to detect early in its early stages because it does not form obvious tumors. Symptoms are often non-specific, which delays diagnosis. Due to its aggressive nature and tendency to metastasize early, the prognosis for this type of cancer is usually worse. This type of cancer is more common in younger patients and is less associated with classic risk factors than the intestinal type but may have stronger genetic links. Due to its infiltrative nature and lack of clear margins, resection requires larger margins, usually greater than 5 cm. In many cases, a more extensive gastric resection, and sometimes a total gastrectomy, is necessary to achieve an adequate tumor-free margin. Removing more lymph nodes and a more thorough assessment of resection margins may also be required [10,11,12,13,14,15].

The immune system plays a crucial role in anticancer surveillance by identifying and eliminating cancer cells. In the context of GC, immune responses can be protective and promote tumor progression. Toll-like receptors (TLRs) are one of the most critical components of the innate immune system and play a key role in recognizing pathogens and triggering the immune response. TLRs are receptors that recognize molecular patterns associated with pathogens (PAMPs) and cell damage (DAMPs) [16,17]. Their activation leads to a signaling cascade that produces pro-inflammatory cytokines such as interleukins (IL) and interferons (IFN), modulating the immune response. TLRs are crucial in recognizing various microorganisms, including bacteria, viruses, fungi, and parasites, which makes them important in the pathogenesis of many infectious and inflammatory diseases [18,19]. The activation of these pathways results in the production of pro-inflammatory cytokines and chemokines, as well as interferons, which can modulate the tumor microenvironment. Increased expression of TLRs and abnormal activation of their signaling pathways may lead to chronic inflammation, promoting carcinogenesis [20]. For this reason, TLRs are constantly being investigated for their therapeutic potential in cancer treatment, including GC [21,22].

In light of our observations and data in the literature, this study aimed to determine the role of selected TLR-2, -3, -4, and -9 in the immunopathogenesis of patients with newly diagnosed and untreated gastric cancer. In our research, we included analyses assessing the percentage of occurrence of the tested TLRs on T and B lymphocyte subpopulations and their concentration in the serum of the examined patients. The studies were performed in the context of comparing patients in individual stages and the most common clinical subtypes of GC, as well as a comparative analysis regarding differences in the gender of recruited patients.

## 2. Results

### 2.1. Characteristics of Patients Recruited to the Study, with Particular Emphasis on TLR-2, -3, -3, and -9; Analyses among Patients with GC and Healthy Volunteers Constituting the Control Group

**Figure 1:**
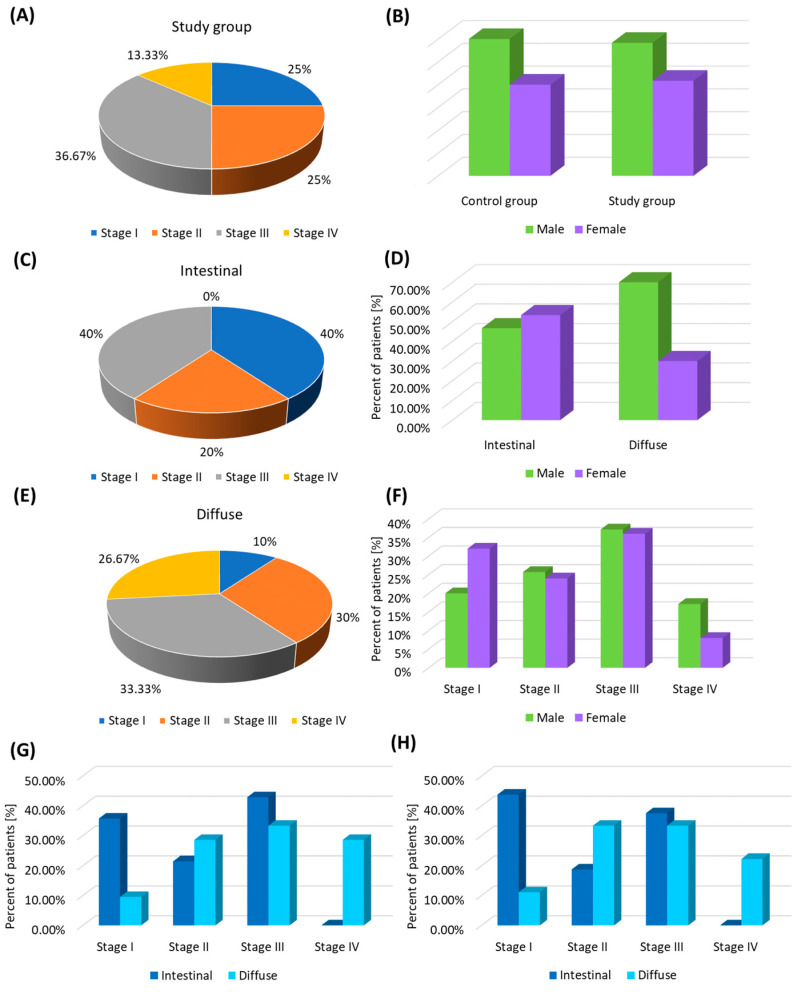
Graphical representation of the differences between GC patients recruited for the study and healthy volunteers constituting the control group. (**A**) Differential stages of GC patients; (**B**) Percentage of patients by gender in each study group; (**C**,**E**) Stage differentiation of GC patients by type; (**D**,**F**) Gender differences in GC patients by type; (**G**) Gender differences in GC patients—males according to stages; (**H**) Gender differences in GC patients—females according to stages.

The study included 60 patients with newly diagnosed gastric cancer (30 patients with intestinal and 30 patients with diffuse type) and 25 healthy volunteers. The average age of patients in the study group was 62.36 ± 10.26 (median 63; range: 40–81 years); the group also included 25 women and 35 men. The different stages of patients with GC are presented in Figure 1. Among the patients recruited to the control group, the mean age was 62.28 ± 9.50 (median: 61; range: 45–78), and the group consisted of 10 women and 15 men. A detailed analysis of basic peripheral blood morphology parameters and immunophenotyping results is presented in Table 1.

**Figure 2:**
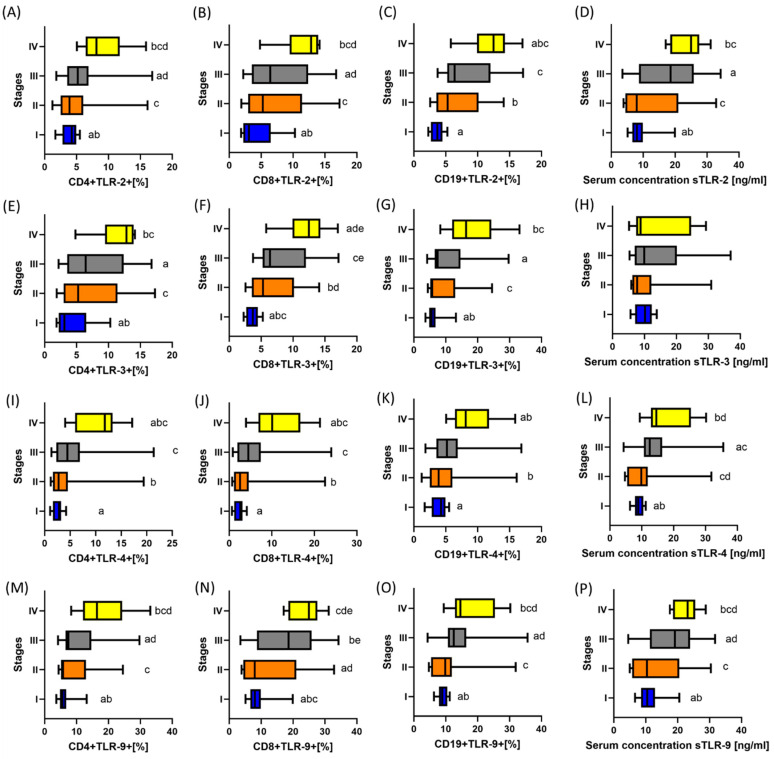
Graphical presentation of the results of assessing the percentage of the tested TLR receptors on individual immune cell subpopulations and the concentration of their soluble forms in the serum of GC patients, taking into account the stages. (**A**–**D**) Results of analyses for TLR-2; (**E**–**H**) Results of analyses for TLR-3; (**I**–**L**) Results of analyses for TLR-4; (**M**–**P**) Results of analyses for TLR-9. Statistical significance between individual stages is marked with lowercase letters on the charts. For convenience, the individual GC stages are marked with colors: blue, stage I; orange, stage II; gray, stage III; and yellow, stage IV.

The analysis of TLRs tested on individual T and B lymphocyte subpopulations showed several significant differences between GC patients and healthy volunteers from the control group. All observed changes were statistically significantly higher in patients with GC compared to the control group. This also applies to the analysis of serum-soluble TLR (sTLR) concentrations between individual patient groups (Table 1). Due to the observed large scatter in the data obtained for patients from the GC group, we decided to check whether these values could be related to the stages to which individual patients from the GC group were classified. As shown in Figure 1A, among all recruited GC patients, 25% were stage I patients, 25% were stage II patients, 36.67% were stage III patients, and 13.33% were stage IV patients. The assessment of the diversity of the studied TLRs, taking into account individual stages, is presented in Supplementary Materials Table S1 and Figure 2.

As we can see in the attached figure and tabular data, in almost each of the analyzed cases, we observed an increase in the average values of the tested TLRs (both from cytometric analyses and an assessment of the concentration of their soluble forms in serum) with an increase in stage advancement in GC patients. A statistical analysis showed that not all of these differences are statistically significant. For CD4+TLR-2+, CD8+TLR-2+, CD4+TLR-4+, CD8+TLR-4+, CD4+TLR-3+, and sTLR9, statistically significant differences were observed between stage I and stage III, stage I and stage IV, stage II and stage IV, and stage III and stage IV (Figure 2). In the case of CD19+TLR-2+ and CD19+TLR-4+, significant changes concerned stages I and stage IV and stage II and stage IV, while for CD19+TLR-3+ and sTLR-2, between stages I and stage III, stage I and stage IV, and stage II and stage IV. In the case of CD8+TLR-3+, statistically significant differences covered almost all analyzed stages except stage II and stage III. For sTLR-4’s concentration, we observed substantial changes between stage I and stage III, stage I and stage IV, stage II and stage III, and stage II and stage IV, while in the case of sTLR-3, we did not observe any statistically significant differences between individual stages.

### 2.2. Comparative Analysis of Tested TLRs in the Context of the Intestinal and Diffuse Type of GC

Due to the recruitment of a comparable group of patients representing the intestinal type and diffuse type of GC, in the next stage of our analyses, we decided to check whether the TLRs examined by our team differed depending on the GC subtype. The patients diagnosed with the intestinal type consisted of 16 women and 14 men (Figure 1C,D), while the patients with the diffuse type consisted of 9 women and 21 men (Figure 1E,F). Both groups showed different stages of disease progression shown in Figure 1G,H.

**Figure 3:**
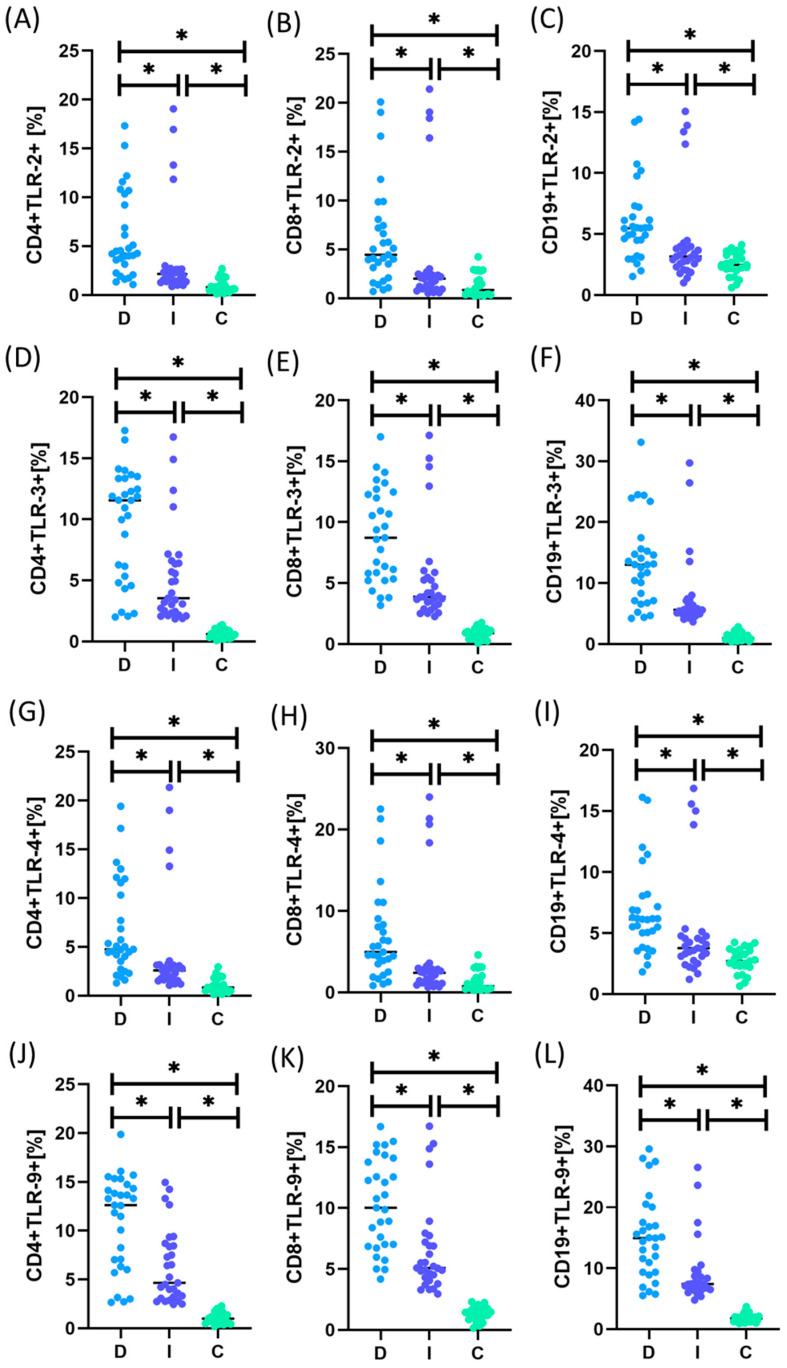
Graphical presentation of the results of assessing the percentage of the tested TLR receptors on individual immune cell subpopulations in patients with GC, including intestinal and diffuse types, compared to people from the control group. (**A**–**C**) Analytical results for TLR-2; (**D**–**F**) Results of analyses for TLR-3; (**G**–**I**) Results of analyses for TLR-4; (**J**–**L**) Results of analyses for TLR-9. Statistical significance between individual groups is marked with * in the charts. For convenience, the different patient groups are color-coded: light blue, patients with diffuse type (D); dark blue, patients with intestinal type (I); and green, patients from the control group (C).

**Figure 4:**
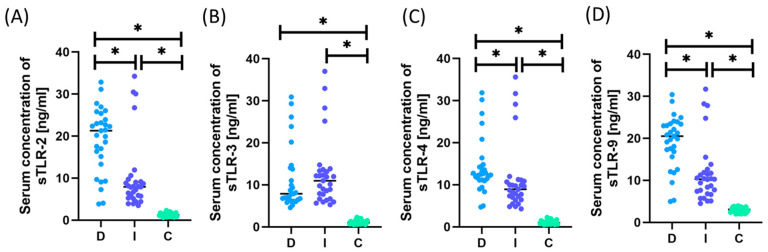
Graphical presentation of the assessment results of the serum concentration of soluble forms of the tested TLR receptors in patients with GC, including intestinal and diffuse types, compared to people from the control group. (**A**) Analytical results for sTLR-2; (**B**) Analytical results for sTLR-3; (**C**) Analytical results for sTLR-4; (**D**) Analytical results for sTLR-9. Statistical significance between individual groups is marked with * in the charts. For convenience, the different patient groups are color-coded: light blue, patients with diffuse type (D); dark blue, patients with intestinal type (I); and green, patients from the control group (C).

Supplementary Materials Table S2 and Figure 3 and Figure 4 present detailed data on the morphology, immunophenotype, and percentage of TLR occurrence in individual immune cell subpopulations and analyses of their serum concentration.

The analysis of the obtained test results showed that both patients with intestinal and diffuse types were characterized by a statistically significantly higher percentage of all tested TLRs on selected T and B lymphocyte subpopulations than the control group. Moreover, the same tendency was maintained in the case of serum concentrations of all analyzed sTLRs. A comparative analysis of both subtypes in patients with GC also showed statistically significant changes in almost all analyzed TLR parameters except sTLR-3. The detailed data presented in Figure 3 show higher values of the tested TLRs in patients with the diffuse type compared to the intestinal type. However, when examining sTLR levels in patients’ serum, higher values were observed only in the case of sTLR-3 for patients with the intestinal type compared to the diffuse type; however, these values were not statistically significant.

### 2.3. Gender Differences in the Course of GC—Can TLR Have an Impact on It?

The next stage of the study concerned the answer to the question of whether there are differences in the percentages of the tested TLRs and their soluble forms in serum between men and women with newly diagnosed GC.

**Figure 5:**
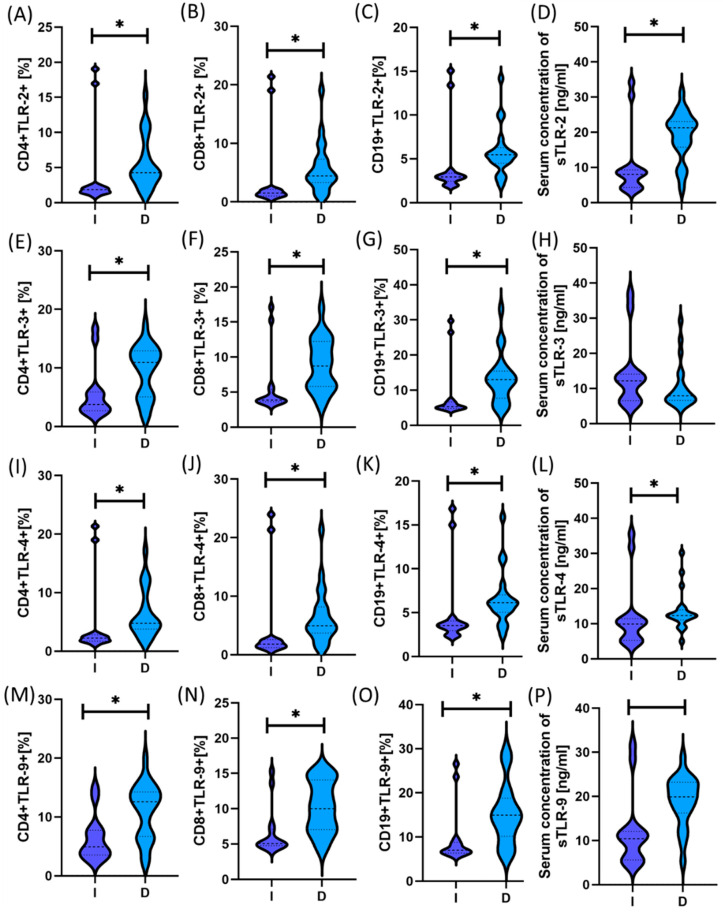
The study includes a graphical summary of the differences in the percentage of tested TLRs and the serum concentration of their soluble forms in men with GC, emphasizing differences in types. For convenience, the different patient groups are color-coded: light blue, patients with diffuse type (D); dark blue, patients with intestinal type (I). Subfigures **A**–**C** show the percentage of CD4+, CD8+, CD19+ lymphocytes expressing TLR2 in patients with diffuse (D) and intestinal (I) type of cancer. Subfigure **D** show serum concentration of soluble forms of TLR2. in patient groups (D) and (I); Subfigure **E**–**G** show the percentage of CD4+, CD8+, CD19+ lymphocytes expressing TLR4 in patients with diffuse (D) and intestinal (I) type of cancer. Subfigure **H** show serum concentration of soluble forms of TLR4 in patient groups (D) and (I); Subfigure **I**–**K** show the percentage of CD4+, CD8+, CD19+ lymphocytes expressing TLR3 in patients with diffuse (D) and intestinal (I) type of cancer. Graphs **L** show serum concentration of soluble forms of TLR3 in patient groups (D) and (I); Subfigure **M**–**O** show percentage of CD4+, CD8+, CD19+ lymphocytes expressing TLR9 in patients with diffuse (D) and intestinal (I) type of cancer. Subfigure **P** shows serum concentration of soluble forms of TLR9 in patient groups (D) and (I). Statis-tical significance between individual groups is marked with * in the charts.

**Figure 6:**
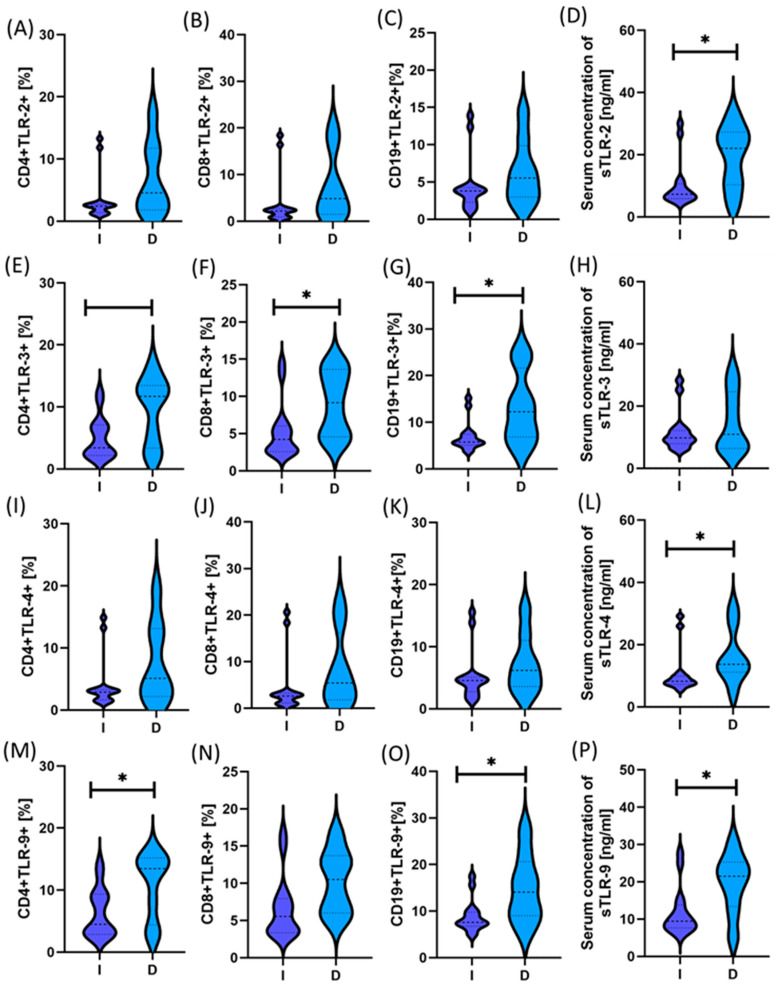
The study includes a graphical summary of the differences in the percentage of tested TLRs and the serum concentration of their soluble forms in females with GC, emphasizing differences in types. For convenience, the different patient groups are color-coded: light blue, patients with diffuse type (D); dark blue, patients with intestinal type (I). Subfigures **A**–**C** show the percentage of CD4+, CD8+, CD19+ lymphocytes expressing TLR2 in patients with diffuse (D) and intestinal (I) type of cancer. Subfigure **D** show serum concentration of soluble forms of TLR2. in patient groups (D) and (I); Subfigure **E**–**G** show the percentage of CD4+, CD8+, CD19+ lymphocytes expressing TLR4 in patients with diffuse (D) and intestinal (I) type of cancer. Subfigure **H** show serum concentration of soluble forms of TLR4 in patient groups (D) and (I); Subfigure **I**–**K** show the percentage of CD4+, CD8+, CD19+ lymphocytes expressing TLR3 in patients with diffuse (D) and intestinal (I) type of cancer. Graphs **L** show serum concentration of soluble forms of TLR3 in patient groups (D) and (I); Subfigure **M**–**O** show percentage of CD4+, CD8+, CD19+ lymphocytes expressing TLR9 in patients with diffuse (D) and intestinal (I) type of cancer. Subfigure **P** shows serum concentration of soluble forms of TLR9 in patient groups (D) and (I). Statis-tical significance between individual groups is marked with * in the charts.

For this purpose, we grouped the results of all GC patients, regardless of stage and type, into two groups based on the gender of the recruited patients. The statistical analyses presented in Supplementary Materials Table S3 does not show statistically significant changes between patients. However, analyses regarding gender differences in analyzing individual types of GC provided us with valuable information (Supplementary Materials Tables S4 and S5). In men, we observed statistically significant differences between the intestinal and diffuse types in almost all analyzed TLRs except sTLR-3 (Figure 5). We noted much fewer differences in the same analyses conducted for women. Here, statistically significant differences concerned CD4+TLR-9+, CD19+TLR-9+, and all tested immune cell populations positively expressing TLR-3; and in the case of serum sTLR concentration, significant changes concerned sTLR-2, -4, and -9 (Figure 6).

### 2.4. Correlation and ROC Curve Analysis

The last stage of the research included performing Spearman’s rank correlation analyses of the examined parameters with the stage of advancement as well as the morphology and immunophenotype of peripheral blood of patients in particular groups. For patients diagnosed with an intestinal type of GC, we recorded 220 positive statistically significant correlations, of which 43 correlations were low; 26 were high, 6 were very high, and 19 were practically full. The latter group includes the following: sTLR2 and sTLR4; sTLR4 and sTLR9; CD19+TLR-9+ [%] and CD19+TLR3+ [%]; CD8+TLR-2+ [%] and CD19+TLR4+ [%]; CD8+TLR4+ [%] and CD19+TLR4+ [%]; CD4+TLR-2+ [%] and CD19+TLR4+ [%]; CD4+TLR4+ [%] and CD19+TLR4+ [%]; sTLR4 and sTLR3;CD8+TLR-2+ [%] and CD19+TLR-2+ [%]; CD19+TLR-2+ [%] and CD8+TLR4+ [%]; CD19+TLR-2+ [%] and CD4+TLR-2+ [%]; CD8+TLR-9+ [%] and CD8+TLR3+ [%]; sTLR2 and sTLR9; CD4+TLR-9+ [%] and CD4+TLR3+ [%]; CD19+TLR-2+ [%] and CD19+TLR4+; CD4+TLR-2+ [%] and CD8+TLR-2+ [%]; CD4+TLR-2+ [%] and CD8+TLR4+ [%]; CD8+TLR-2+ [%] and CD4+TLR4+ [%]; CD4+TLR4+ [%] and CD8+TLR4+ [%] (Supplementary Materials Table S6). In the case of patients with the diffuse type, we recorded 260 statistically significant correlations, 12 of which were negative correlations of a low, moderate nature. Among the positive correlations, 20 had low correlations, 36 had moderate correlations, 56 had high correlations, 103 had very high correlations, and 32 had practically full correlations. The latter group includes: Neutrophils and sTLR-2; Lymphocytes and CD4+TLR-2+ [%]; Lymphocytes and sTLR-2; Lymphocytes and CD4+TLR-4+ [%]; CD19+TLR-2+ [%] and CD8+TLR-3+ [%]; CD19+TLR-4+ [%] and CD8+TLR-3+ [%]; White blood cells and CD19+TLR-2+ [%]; Neutrophils and CD19+TLR-2+ [%]; White blood cells and CD19+TLR-4+ [%]; Neutrophils and CD19+TLR-4+ [%]; CD8+TLR-2+ [%] and CD19+TLR-4+ [%]; CD8+TLR-4+ [%] and CD19+TLR-4+ [%]; White blood cells and Lymphocytes; Lymphocytes and CD19+TLR-2+ [%]; Lymphocytes and CD19+TLR-4+ [%]; CD4+TLR-2+ [%] and CD19+TLR-4+ [%]; CD8+TLR-2+ [%] and CD19+TLR-2+ [%]; CD19+TLR-2+ [%] and CD8+TLR-4+ [%]; CD4+TLR-4+ [%] and CD19+TLR-4+ [%]; CD4+TLR-2+ [%] and CD19+TLR-2+ [%]; CD4+TLR-9+ [%] and CD4+TLR-3+ [%]; Neutrophils and Lymphocytes; CD19+TLR-2+ [%] and CD4+TLR-4+ [%]; White blood cells and Neutrophils; sTLR-2 and sTLR-9 [ng/mL]; CD8+TLR-9+ [%] and CD8+TLR-3+ [%]; CD19+TLR-9+ [%] and CD19+TLR-3+ [%]; CD8+TLR-2+ [%] and CD4+TLR-4+ [%]; CD4+TLR-4+ and CD8+TLR-4+ [%]; CD4+TLR-2+ [%] and CD8+TLR-2+ [%]; CD4+TLR-2+ [%] and CD8+TLR-4+ [%]; CD4+TLR-2+ [%] and CD4+TLR-4+ [%] (Supplementary Materials Table S7).

The correlation analysis of the tested parameters between men diagnosed with GC showed the presence of 251 statistically significant correlations, of which only 4 were low negative correlations, while 247 were positive correlations. Among the positive correlations, 17 were low correlations; 56 were moderates; 61 were tall; 88 were very high, and 25 were practically full. The latter include the following: CD4+TLR-2+ [%] and CD4+TLR-3+ [%]; CD4+TLR-3+ [%] and CD4+TLR-4+ [%]; sTLR-4 and sTLR-9 [ng/mL]; sTLR-2 and sTLR-4; CD19+TLR-4+ [%] and CD4+TLR-3+ [%]; CD19+TLR-2+ [%] and CD4+TLR-3+ [%]; Neutrophils and Monocytes; White blood cells and Neutrophils; CD8+TLR-4+ [%] and CD19+TLR-4+ [%]; CD8+TLR-2+ [%] and CD19+TLR-4+ [%]; CD4+TLR-2+ [%] and CD19+TLR-4+ [%]; CD19+TLR-4+ [%] and CD4+TLR-4+ [%]; CD19+TLR-2+ [%] and CD8+TLR-4+ [%]; CD8+TLR-2+ [%] and CD19+TLR-2+ [%]; CD4+TLR-2+ [%] and CD19+TLR-2+ [%]; CD19+TLR-2+ [%] and CD4+TLR-4+ [%]; CD8+TLR-9+ [%] and CD8+TLR-3+ [%]; CD4+TLR-9+ [%] and CD4+TLR-3+ [%]; sTLR-2 and sTLR-9 [ng/mL]; CD4+TLR-2+ [%] and CD8+TLR-2+ [%]; CD8+TLR-2+ [%] and CD4+TLR-4+ [%]; CD4+TLR-2+ [%] and CD8+TLR-4+ [%]; CD8+TLR-4+ [%] and CD4+TLR-4+ [%]; CD19+TLR-2+ [%] and CD19+TLR-4+ [%]; CD8+TLR-2+ [%] and CD8+TLR-4+ [%] (Supplementary Materials Table S8). In the case of women diagnosed with GC, we noted 246 statistically significant correlations (5 moderately negative and 241 positive correlations). In the group of positive correlations, we noted 9 low correlations, 53 moderate, 126 high, 28 very high, and 25 practically complete. The last group includes the following: CD8+TLR-9+ [%] and CD19+TLR-9+ [%]; CD19+TLR-9+ [%] and CD8+TLR-3+ [%] and CD19+TLR-3+ [%]; White blood cells and sTLR-2 and sTLR-4 ratio; sTLR-4 and sTLR-9 [ng/mL]; CD8+TLR-2+ [%] and CD19+TLR-4+ [%]; sTLR-2 and sTLR-9 [ng/mL]; CD4+TLR-2+ [%] and CD4+TLR-4+ [%] and CD19+TLR-4+ [%]; CD8+TLR-2+ [%] and CD19+TLR-2+ [%]; -2+ [%] and CD19+TLR-2+ [%]; CD19+TLR-9+ [%] and CD19+TLR-3+ [%]; -4+ [%]; CD4+TLR-9+ [%] and CD4+TLR-3+ [%]; -2+ [%] and CD4+TLR-4+ [%]; CD8+TLR-4+ [%] and CD4+TLR-4+ [%]; CD4+TLR-2+ [%] and CD8+TLR-2+ [%]; CD4+TLR-2+ [%] and CD8+TLR-4+ [%]; CD4+TLR-2+ [%] and CD4+TLR-4+ [%] (Supplementary Materials Table S9).

**Figure 7:**
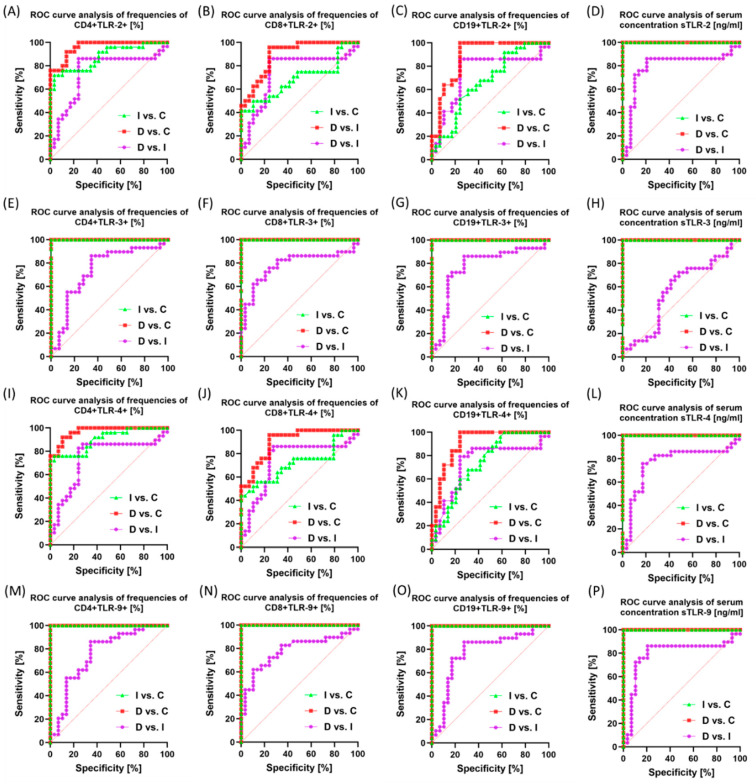
Graphical representation of ROC curves for patients with intestinal and diffuse types compared to control patients. (**A**–**D**) Results obtained for TLR-2 analyses; (**E**–**H**) Results obtained for TLR-3 analyses; (**I**–**L**) Results obtained for TLR-4 analyses; (**M**–**P**) Results obtained for TLR-9 analyses. For convenience, the analysis between the diffuse type and the intestinal type is marked in purple, between the diffuse type and the control group, and in green between the intestinal type and the control group. Abbreviations: I—intestinal type; D—diffuse type; C—control group.

In the next stage of our analyses, we decided to check whether the TLR results obtained by our team could constitute a potential biomarker molecule. For this purpose, we analyzed ROC curves between individual GC subtypes and the control group (Figure 7 and Supplementary Materials Table S10), between individual GC stages (Supplementary Materials Table S11), and between men and women in individual GC subtypes (Supplementary Material Table S12).

In the first analyzed case, a sensitive and specific biomarker molecule for patients with both intestinal and diffuse type about patients from the control group may be sTLR-2 or sTLR4 and CD4+TLR-3; CD8+TLR-3+; CD19+TLR-3+ and sTLR3 as well as CD4+TLR-9; CD8+TLR-9+; CD19+TLR-9+ and sTLR-9 (Figure 7 and Supplementary Materials Table S10).

Unfortunately, none of the analyzed molecules showed the same potential when comparing both tested GC subtypes. However, the analyses conducted by our team between individual stages of GC (Supplementary Materials Table S11) and between men and women in individual GC subtypes (Supplementary Material Table S12) did not indicate any of the tested TLRs as molecules with high sensitivity and specificity.

## 3. Discussion

TLR expression in malignant tumors, including gastric cancer, plays a key role in regulating the immune response and in inflammatory processes that may influence tumor development and progression. In our work, we focused on examining differences in the expression of TLR-2, -3, -4, and -9 in two subtypes of gastric cancer, intestinal and diffuse, to better understand their potential impact on the course of the disease.

Among the available literature, TLR-4 and TLR-2 are particularly interesting, as they may be activated by chronic inflammation caused by *H. pylori* infection. TLR-4, which recognizes bacterial lipopolysaccharide (LPS), is often overexpressed in GC cells. Its activation leads to increased secretion of pro-inflammatory cytokines such as IL-6, IL-1β, and TNF-α, which may promote inflammation and support a pro-tumor environment [23,24,25,26,27]. Studies indicate that TLR4 signaling can promote cancer cell proliferation, invasion, and resistance to apoptosis. TLR2, which recognizes cell wall components of Gram-positive bacteria, also plays a role in the pathogenesis of GC. Like TLR-4, activation of TLR-2 leads to the production of pro-inflammatory cytokines that may support cancer processes [25,28,29,30,31]. Our previous studies also showed an essential role of TLR2 in the progression of this cancer [25]. Moreover, TLR-2 is associated with lymph node metastases and distant metastases; its high expression may also contribute to a worse prognosis [31,32,33].

A study conducted by Belén et al. showed that tumor cells had high expression of TLR-3, TLR-4, and TLR-9 compared to stromal cells and that high TLR-3 expression was significantly associated with poorer overall survival in patients with resectable tumors [34]. Some studies suggest that TLR-3 may be a diagnostic marker for gastric adenocarcinoma [35,36]. However, Zheng’s work suggests that TLR-3 may have both pro- and anti-tumor effects, depending on the context of the tumor microenvironment [37]. Fernandez-Garcia et al., however, did not find a relationship between the expression of TLR-4 and TLR-9 in tumors and clinicopathological factors in patients with GC [35]. Research by two teams, Wang et al. and Yap et al., showed that people from the Chinese population who have the TLR-9-1486C allele in their genotype are characterized by an increased risk of GC and worse prognosis in the course of this disease [36,37]. The team of Kasurinen et al. showed that high expression of TLR-1, TLR-2, TLR-4, TLR-5, TLR-7, and TLR-9 receptors was observed in intestinal GC samples. In particular, high TLR5 levels have been identified as a biomarker of better prognosis, especially in patients with stage II disease [22]. Eskuri et al., in their analyses, showed that high expression of TLR-6 may contribute to a better 5-year prognosis compared to the group characterized by low expression. At the same time, TLR-1, TLR-4, and TLR-5 do not seem to be appropriate prognostic factors [35,38,39,40].

Although the studies presented in our study show statistically significant changes between the examined patients, suggesting the involvement of the tested TLRs in the immunopathogenesis of GC, they cover only a small fragment of the immunological landscape of the disease. One of the main limitations of our study was the relatively small number of participants. The small sample size may limit the generalizability of the results to the broader population of gastric cancer patients. It should also be mentioned that the study mainly included patients from a specific geographic region and specific ethnic background, which may affect the results and their generalization to other populations. The studies presented in this publication focused on patients with newly diagnosed and untreated gastric cancer, which is an essential aspect of the analysis of the pathogenesis of GC, but the lack of long-term follow-up makes it impossible to assess the impact of TLR expression on the course of the disease and patient survival. We hope that in the future, it will be possible to take measures to monitor the percentage of TLR occurrence in these patients, taking into account the treatment used and the remission time. TLRs’ role in gastric cancer immunopathogenesis is complex. It may be modulated by many other factors, such as the tumor microenvironment, other signaling pathways, and individual differences in patient immune responses. Our study did not consider all these variables or include the analysis of all TLRs, which is undoubtedly a limitation of this study. Considering these limitations is crucial when interpreting the results of our study and planning future research in this area.

To overcome the limitations of a small sample size, future studies should include larger and more diverse patient populations, which will increase the reliability and generalizability of the results. Including long-term follow-up of patients will allow for the assessment of the impact of TLRs on the course of disease and survival, providing valuable information about their potential prognostic role. Further studies should also include more complex analyses that include various biological and clinical factors affecting TLR function. Advanced molecular and bioinformatic techniques may help to better understand the mechanisms of action of these receptors in the context of gastric cancer. We strongly believe that understanding the role of TLRs in the immunopathogenesis of gastric cancer may lead to developing new therapeutic strategies that will be more effective and personalized. The development of therapies targeting specific TLRs may open new possibilities in the treatment of gastric cancer, improving treatment outcomes and the quality of life of patients.

## 4. Materials and Methods

### 4.1. Patient Characteristics

The study included 60 patients with newly diagnosed gastric cancer, histopathologically confirmed based on endoscopic biopsy results, and 25 healthy volunteers. Confirming the diagnosis of GC involved several steps. The patients came from the Department of General and Oncological Surgery, Faculty of Medicine, Medical University of Lodz. The process began with a medical history and physical examination, where the referring physician took information about the patient’s symptoms, such as abdominal pain, loss of appetite, weight loss, nausea, vomiting, and difficulty swallowing, as well as medical history and risk factors, such as smoking, tobacco, diet. Then, basic laboratory tests were performed, including blood counts, liver and kidney function tests, and determination of tumor markers such as CA 19-9 and CEA. The key element of the diagnostics was endoscopy of the upper gastrointestinal tract (gastroscopy), which allows for direct visualization of the inside of the stomach using a flexible endoscope equipped with a camera. During gastroscopy, the doctor identified pathological changes, such as tumors and ulcers, and took biopsies from suspicious areas for further histopathological analysis. Histopathological analysis of the tissue obtained during the biopsy allowed for confirming the presence of cancer cells and determining the type of cancer (adenocarcinoma) and the stage of advancement. Additional imaging tests were performed to assess the spread of the cancer and its stage. Computed tomography (CT) of the abdominal cavity and pelvis allows for the assessment of the size of the tumor, its location, and possible metastases to lymph nodes and other organs. In some cases, magnetic resonance imaging (MRI) was performed and was used in addition to CT for better evaluation.

Inclusion criteria included patients with newly diagnosed gastric cancer, confirmed histopathologically, who had not previously received any anticancer treatment, such as chemotherapy, radiotherapy, or immunotherapy. Patients must be over 18 years of age and give written consent to participate in the study after being fully informed about its goals, procedures, and potential risks. Exclusion criteria include patients with other active cancers or a history of other cancers, with the exception of skin cancer in situ or cured cervical cancer. Also excluded were patients with active autoimmune diseases or severe chronic diseases affecting the immune system, as well as patients with active viral, bacterial, or fungal infections requiring antibiotic or antiviral therapy. Patients using immunosuppressive drugs or high-dose corticosteroids in the last 6 months, with the exception of low doses used to treat chronic diseases, were also excluded. Additionally, patients with severe comorbidities that could affect the study results or be life-threatening, such as heart failure, renal failure, liver failure, decompensated diabetes, or severe respiratory disease, were not allowed to participate in the study. Pregnant or breastfeeding women were excluded, as were patients participating in other clinical trials in the last 30 days before study initiation. These criteria were intended to ensure the safety of study participants and to obtain reliable and consistent research results.

### 4.2. Immunophenotyping

**Figure 8:**
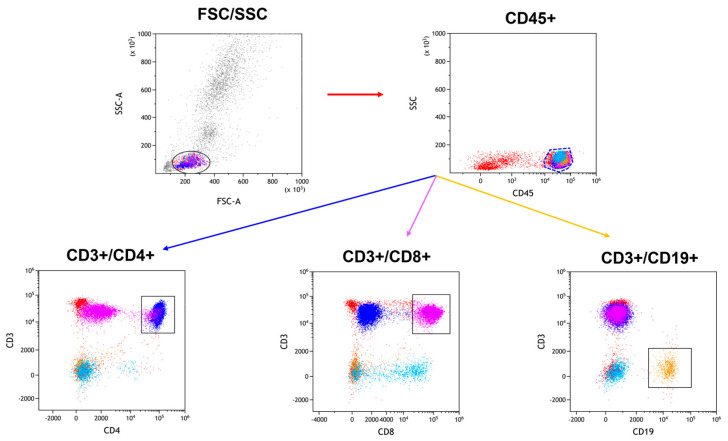
Example of a gating strategy for assessing the CD3+/CD4+ (dark blue), CD3+/CD8+ (purple), CD3+/CD19+ (orange) subpopulations.

**Figure 9:**
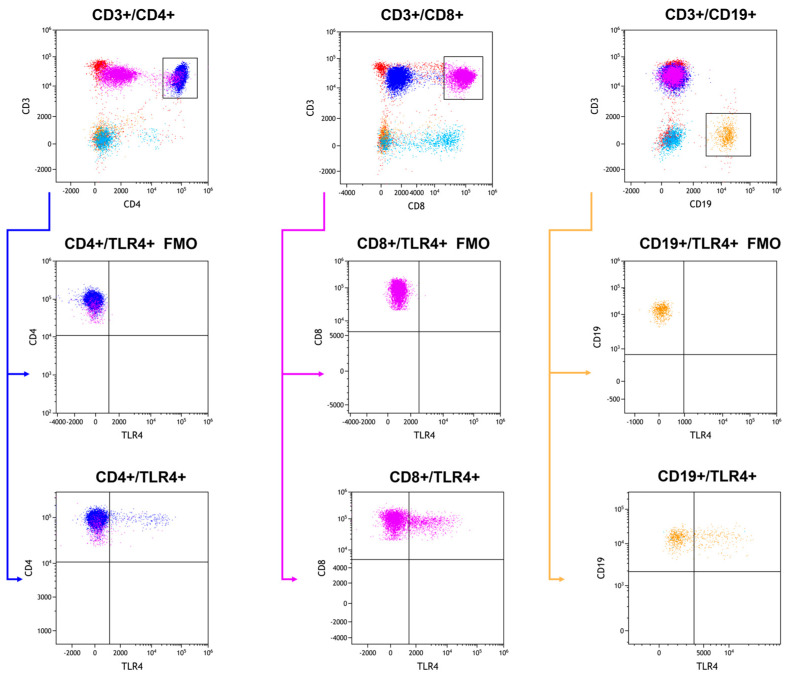
Example of a gating strategy for assessing TLR4 on subpopulations CD3+/CD4+ (dark blue), CD3+/CD8+ (purple), and CD3+/CD19+ (orange).

Whole-blood samples from patients were incubated with monoclonal antibodies conjugated with fluorochromes targeting specific human antigens (e.g., anti-CD45 FITC, anti-CD3B V510, anti-CD4BV650, anti-CD8 BV605, anti-CD19 PERCP CY 5.5, anti-CD16 +56 APC-CY7, anti-TLR2 APC, anti-TLR4 PE, anti-TLR7 PE, anti-TLR8 APC, anti-TLR3 PE, and anti-TLR9 APC) from Biolegend. In connection with the use of several fluorochromes excited by violet light, in order to obtain better stability and signals, the BD Horizon™ Brilliant Stain Buffer (BD, Franklin Lakes, NJ, USA) was added to the samples. The samples were then treated with lysis buffer to remove erythrocytes and washed with BD Pharmingen™ Stain Buffer (BSA). For permeabilization and internal TLR receptor labeling, the BD Cytofix/Cytoperm™ Fixation/Permeabilization Kit was utilized. The samples were analyzed using the CytoFLEX LX instrument (Beckman Coulter, Indianapolis, IN, USA), and data analysis was conducted with the Kaluza Analysis program v 2.1 (sample analysis in Figure 8 and Figure 9). Device calibration was ensured using CytoFLEX Ready to Use Daily QC Fluorosphere reagents (Beckman Coulter, Indianapolis, IN, USA).

### 4.3. Determinations of Soluble Forms of Tested TLRs (sTLR)

To assess the levels of TLR-2, TLR-3, TLR-4, and TLR-9 receptors in patients’ serum, ELISA (Enzyme-Linked Immunosorbent Assay) tests were used. Commercial ELISA kits for TLR-2 (15.6–1000 pg/mL), TLR-3 (31.2–2000 pg/mL), TLR-4 (15.6–1000 pg/mL), and TLR-9 (31.2–2000 pg/mL) were purchased from R&D Systems. ELISA plates were coated with specific antibodies directed against the tested receptors. The absorbance in each well was measured on a Victor 4.0 microplate reader (PerkinElmer) using compatible software. The results were analyzed in relation to the calibration curve obtained from the standards, which allowed for quantifying the concentrations of TLR-2, TLR-3, TLR-4, and TLR-9 in serum samples.

### 4.4. Statistics

The Shapiro–Wilk test was used to check the normality of data distribution. Although this is a parametric test, it was used to verify that the data met the assumptions required by parametric tests, which justified the use of nonparametric methods for data that did not meet these assumptions. The Mann–Whitney U test was used to compare two independent groups. This test assesses whether there are statistically significant differences between the medians of two groups, which is especially useful for small samples and data that are not normally distributed. To compare more than two independent groups, the Kruskal–Wallis test was used, which is an extension of the Mann–Whitney U test and allows for assessing whether at least one of the groups differs significantly in terms of the median from the others. The Kruskal–Wallis test is the non-parametric equivalent of one-way analysis of variance (ANOVA). Additionally, a modification of the Kruskal–Wallis test with Bonferroni transformation was used to correct the level of significance in multiple group comparisons, which increases the accuracy of the results. The Statistica package was used to analyze the data, and the results were visualized using GraphPad software. To visualize the data, box plots were used to present the distribution of data, median, quartiles, and outliers in different groups, and dot plots were used to present individual point data in groups, which makes it easier to understand the distribution of data in the subject populations. Additionally, violin plots were used, which combine the features of box plots and density plots, enabling the visualization of the probability distribution of data in different groups. Violin plots are particularly useful for showing the full distribution of data and identifying potential differences between groups.

## 5. Conclusions

Our research results indicate that TLR-2, -3, -4, and -9 play an important role in the immunopathogenesis of gastric cancer. The differential expression of these receptors in different subpopulations of T and B lymphocytes suggests their significant impact on the body’s immune response to cancer. Our analyses showed significant differences in TLR expression depending on the stage of gastric cancer and its clinical subtypes, which may be of key importance for the diagnosis and prognosis of the course of the disease. We found that early-stage gastric cancer and specific clinical subtypes exhibit unique TLR expression patterns, highlighting their potential role as biomarkers. Although our studies did not reveal significant differences in TLR expression between sexes, we observed significant changes within each sex depending on the analyzed gastric cancer subtype. This may suggest that the patient’s gender may influence the immune response to the tumor, which may affect personalized therapeutic approaches.

These conclusions, although providing important information on the role of TLRs in gastric cancer, require further verification in studies with larger groups of patients and longer observation periods. Additional studies should also use more advanced analytical techniques to further investigate the mechanisms of action of TLRs and their impact on the immunopathogenesis of gastric cancer. Differential TLR expression can be used as a biomarker in the diagnosis and monitoring of the progression of gastric cancer. Furthermore, these receptors may represent targets for new, more effective therapeutic strategies, leading to improved treatment outcomes for gastric cancer patients.

## Figures and Tables

**Table 1:** Tabular summary of the diversity of morphology and immunophenotype of peripheral blood in patients with GC recruited for the study and healthy volunteers constituting the control group, taking into account statistical significance.

Parameter	GC Patients Group		Control Group		*p*-Value
	Mean ± SD	Median (Range)	Mean ± SD	Median (Range)	
Age	62.36 ± 10.26	63.00 (40.00–81.00)	62.28 ± 9.50	61.00 (45.00–78.00)	0.786
Neutrophils [10^3^/mm^3^]	4.43 ± 1.07	4.23 (2.47–6.10)	4.21 ± 0.86	4.44 (2.20–5.87)	0.388
Monocytes [10^3^/mm^3^]	0.46 ± 0.12	0.46 (0.22–0.70)	0.45 ± 0.12	0.47 (0.10–0.75)	0.886
Lymphocytes [10^3^/mm^3^]	2.16 ± 0.46	2.10 (1.31–3.14)	2.57 ± 0.56	2.61 (1.57–4.10)	0.002 *
T lymphocytes CD3+ [%]	69.55 ± 5.04	70.71 (58.89–79.79)	71.59 ± 2.68	71.28 (65.34–76.70)	0.158
B lymphocytes CD19+ [%]	10.86 ± 2.37	10.78 (7.31–16.30)	11.96 ± 2.33	11.75 (7.62–16.82)	0.061
T lymphocytes CD3+CD4+ [%]	38.33 ± 3.36	37.50 (31.76–47.08)	40.52 ± 3.13	40.44 (34.97–46.35)	0.006 *
T lymphocytes CD3+CD8+ [%]	27.82 ± 4.07	27.54 (19.83–36.44)	30.30 ± 3.08	30.49 (24.19–35.78)	0.006 *
T lymphocytes ratio CD3+CD4+/T CD3+CD8+	1.36 ± 0.25	1.33 (0.96–2.02)	1.36 ± 0.23	1.28 (1.03–1.90)	0.957
T lymphocytes CD4+TLR-2+ [%]	4.85 ± 4.66	2.64 (0.89–19.05)	0.95 ± 0.66	0.81 (0.13–2.70)	0.000 *
T lymphocytes CD8+TLR-2+ [%]	5.08 ± 5.67	2.43 (0.53–21.41)	1.30 ± 1.13	0.71 (0.24–4.24)	0.000 *
B lymphocytes CD19+TLR-2+ [%]	5.18 ± 3.58	3.97 (1.00–15.05)	2.55 ± 0.93	2.48 (0.61–4.12)	0.000 *
T lymphocytes CD4+TLR-3+ [%]	7.45 ± 4.74	6.21 (1.86–17.28)	0.64 ± 0.33	0.60 (0.09–1.35)	0.000 *
T lymphocytes CD8+TLR-3+ [%]	7.22 ± 4.24	5.64 (2.25–17.12)	0.85 ± 0.41	0.90 (0.07–1.76)	0.000 *
B lymphocytes CD19+TLR-3+ [%]	10.51 ± 7.08	7.11 (3.64–33.12)	1.21 ± 0.64	0.95 (0.43–2.80)	0.000 *
T lymphocytes CD4+TLR-4+ [%]	5.52 ± 5.17	3.16 (1.07–21.34)	1.02 ± 0.71	0.85 (0.14–2.94)	0.000 *
T lymphocytes CD8+TLR-4+ [%]	5.76 ± 6.31	2.91 (0.64–23.98)	1.40 ± 1.22	0.77 (0.26–4.62)	0.000 *
B lymphocytes CD19+TLR-4+ [%]	5.93 ± 3.94	4.77 (1.20–16.86)	2.73 ± 0.98	2.70 (0.66–4.24)	0.000 *
T lymphocytes CD4+TLR-9+ [%]	8.66 ± 4.84	7.88 (2.45–19.86)	1.02 ± 0.54	0.99 (0.19–2.28)	0.000 *
T lymphocytes CD8+TLR-9+ [%]	8.44 ± 4.13	7.03 (2.95–16.72)	1.35 ± 0.57	1.44 (0.15–2.31)	0.000 *
B lymphocytes CD19+TLR-9+ [%]	12.16 ± 6.65	9.36 (4.79–29.57)	1.84 ± 0.68	1.77 (0.94–3.68)	0.000 *
TLR-2 serum concentration [ng/mL]	14.75 ± 9.13	10.19 (3.49–34.24)	1.32 ± 0.44	1.21 (0.62–2.27)	0.000 *
TLR-3 serum concentration [ng/mL]	12.17 ± 7.76	9.19 (4.59–37.03)	1.15 ± 0.51	1.03 (0.54–2.29)	0.000 *
TLR-4 serum concentration [ng/mL]	12.77 ± 7.48	11.10 (4.29–35.61)	1.10 ± 0.49	0.99 (0.52–2.21)	0.000 *
TLR-9 serum concentration [ng/mL]	15.50 ± 7.62	13.23 (4.53–31.70)	2.99 ± 0.58	3.08 (2.03–3.92)	0.000 *

* statistically significant results.

## Data Availability

All the necessary information regarding the preparation of this work is available by written request to the corresponding author.

## Supplementary Materials

The following supporting information can be downloaded at: https://www.mdpi.com/article/10.3390/ijms25179264/s1.
