# Supplementary material for: New Horizons in the Diagnosis of Gastric Cancer: The Importance of Selected Toll-like Receptors in Immunopathogenesis Depending on the Stage, Clinical Subtype, and Gender of Newly Diagnosed Patients
Source: Int J Mol Sci. 2024 Aug 27;25(17):9264. doi: 10.3390/ijms25179264 (PMC11394694; doi:10.3390/ijms25179264)
Supplement: Supplementary file 1 [file ijms-25-09264-s001.zip › ijms-3146014-supplementary.pdf]

Supplementary Materials Table S1. Tabular summary of the diversity of peripheral blood morphology and immunophenotype and the percentage of TLRs tested in patients with GC recruited for the study

**Parameter**	**Stage I**		**Stage II**		**Stage III**		**Stage IV**		**p-value**					
	**Mean±SD**	**Median (Range)**	**Mean±SD**	**Median (Range)**	**Mean±SD**	**Median (Range)**	**Mean±SD**	**Median (Range)**	1 vs. 2	1 vs. 3	1 vs. 4	2 vs. 3	2 vs. 4	3 vs. 4
Age	63.54±9.43	67.00 (49.00-76.00)	60.00±11.87	60.50 (41.00-81.00)	65.84±7.26	65.00 (51.00-76.00)	60.00±10.93	60.00 (40.00-77.00)	0.619	0.734	0.547	0.066	0.976	0.238
White blood cells $[10^3/mm^3]$	6.72±0.72	6.76 (5.39-7.70)	7.44±1.17	7.44 (5.79-10.20)	7.65±0.99	7.57 (5.99-9.57)	9.18±0.52	9.15 (8.22-10.12)	0.144	0.024*	0.000*	0.441	0.002*	0.000*
Neutrophils $[10^3/mm^3]$	3.83±0.72	3.86 (2.62-4.85)	4.24±1.03	3.98 (2.71-6.10)	4.44±1.07	5.05 (2.47-5.84)	5.78±0.28	5.83 (5.11-6.09)	0.374	0.126	0.000*	0.612	0.003*	0.009*
Monocytes $[10^3/mm^3]$	0.41±0.12	0.36 (0.29-0.70)	0.45±0.10	0.44 (0.33-0.68)	0.45±0.12	0.49 (0.22-0.66)	0.58±0.06	0.58 (0.46-0.67)	0.199	0.404	0.006*	0.832	0.005*	0.001*
Lymphocytes $[10^3/mm^3]$	2.00±0.44	1.96 (1.31-3.05)	2.17±0.38	2.10 (1.68-3.14)	2.07±0.44	1.97 (1.42-3.06)	2.69±0.25	2.69 (2.25-3.10)	0.215	0.821	0.001*	0.461	0.004*	0.025*
T lymphocytes CD3+ [%]	67.54±4.55	66.80 (59.45-73.99)	70.00±4.12	70.64 (61.82-75.30)	69.35±5.27	70.67 (58.89-79.40)	73.29±3.76	74.42 (66.17-79.79)	0.110	0.305	0.006*	0.707	0.172	0.418
B lymphocytes CD19+ [%]	10.69±2.67	10.71 (7.31-16.30)	11.02±2.41	9.95 (8.24-15.07)	10.44±1.81	10.45 (7.58-14.27)	11.75±2.87	11.62 (8.52-16.14)	0.589	0.970	0.414	0.612	0.610	0.025*
T lymphocytes CD3+CD4+ [%]	36.07±2.66	36.09 (31.76-42.10)	39.39±2.98	38.90 (35.17-46.59)	38.32±2.71	37.40 (33.11-42.32)	40.80±3.81	40.23 (34.53-47.08)	0.007*	0.037*	0.013*	0.350	0.383	0.106
T lymphocytes CD3+CD8+ [%]	29.23±3.33	29.54 (23.67-34.90)	28.50±4.06	28.81 (21.39-34.87)	27.64±4.53	27.11 (19.83-36.44)	24.77±1.67	24.10 (22.80-28.08)	0.779	0.404	0.003*	0.461	0.052	0.004*
T lymphocytes ratio CD3+CD4+/T CD3+CD8+	1.18±0.14	1.19 (0.96-1.42)	1.37±0.25	1.31 (1.06-1.89)	1.36±0.21	1.35 (1.03-1.85)	1.66±0.23	1.61 (1.42-2.02)	0.028*	0.030*	0.000*	1.000	0.016*	0.039*
T lymphocytes CD4+TLR-2+ [%]	2.09±0.83	2.33 (0.89-3.75)	4.15±4.46	2.27 (0.99-17.32)	5.63±5.32	3.97 (1.08-19.05)	9.33±3.68	10.53 (3.60-15.30)	0.475	0.024*	0.000*	0.172	0.005*	0.039*
T lymphocytes CD8+TLR-2+ [%]	1.86±0.95	2.13 (0.53-3.67)	4.00±5.07	2.10 (0.58-20.10)	6.30±6.71	4.00 (0.69-21.41)	10.01±4.96	8.98 (3.50-19.03)	0.559	0.030*	0.000*	0.151	0.004*	0.034*
B lymphocytes CD19+TLR-2+ [%]	3.29±1.04	3.53 (1.39-4.92)	4.43±3.37	3.24 (1.00-14.40)	5.98±4.18	4.63 (1.52-15.05)	8.19±2.98	7.26 (4.52-14.19)	0.714	0.065	0.000*	0.205	0.005*	0.075
T lymphocytes CD4+TLR-3+ [%]	4.17±2.49	3.07 (1.86-10.30)	7.11±4.81	5.24 (1.89-17.28)	8.26±4.76	6.41 (2.18-16.74)	11.57±3.05	12.82 (4.80-14.13)	0.503	0.024*	0.000*	0.172	0.005*	0.039*
T lymphocytes CD8+TLR-3+ [%]	3.68±0.96	3.68 (2.25-5.25)	6.54±3.83	5.27 (2.56-14.09)	8.36±4.09	6.38 (3.73-17.12)	12.01±3.16	12.49 (5.80-17.01)	0.589	0.030*	0.000*	0.151	0.004*	0.034*

B lymphocytes CD19+TLR-3+ [%]	6.06±2.33	5.58 (3.64-13.14)	9.60±6.39	5.78 (4.38-24.53)	11.27±6.88	7.43 (4.20-29.72)	18.49±7.62	16.34 (8.33-33.12)	0.779	0.071	0.000*	0.205	0.005*	0.084
T lymphocytes CD4+TLR-4+ [%]	2.49±0.95	2.80 (1.07-4.20)	4.74±4.95	2.73 (1.19-19.40)	6.38±5.91	4.45 (1.30-21.34)	10.45±4.12	11.79 (4.03-17.14)	0.503	0.024*	0.000*	0.172	0.005*	0.039*
T lymphocytes CD8+TLR-4+ [%]	2.21±1.10	2.55 (0.64-4.11)	4.55±5.65	2.51 (0.70-22.51)	7.12±7.48	4.48 (0.83-23.98)	11.21±5.55	10.05 (3.92-21.31)	0.589	0.030*	0.000*	0.151	0.004*	0.034*
B lymphocytes CD19+TLR-4+ [%]	3.91±1.21	4.24 (1.67-5.51)	5.09±3.72	3.89 (1.20-16.13)	6.80±4.61	5.19 (1.82-16.86)	9.17±3.33	8.13 (5.06-15.89)	0.779	0.071	0.000*	0.205	0.005*	0.084
T lymphocytes CD4+TLR-9+ [%]	5.36±2.97	4.04 (2.45-11.84)	8.39±4.89	6.90 (2.49-19.86)	9.36±4.67	8.44 (2.87-15.53)	12.95±3.10	13.71 (6.32-16.10)	0.144	0.016*	0.000*	0.441	0.027*	0.022*
T lymphocytes CD8+TLR-9+ [%]	4.77±1.18	4.84 (2.95-6.91)	7.77±3.80	6.94 (3.37-15.20)	9.57±3.69	8.39 (4.90-16.72)	13.38±2.74	14.36 (7.63-16.69)	0.028*	0.000*	0.000*	0.117	0.004*	0.008*
B lymphocytes CD19+TLR-9+ [%]	7.81±2.58	7.34 (4.79-15.10)	11.36±6.38	7.60 (5.76-26.91)	12.68±5.79	9.77 (5.52-26.54)	20.36±6.74	18.79 (10.96-29.57)	0.156	0.007*	0.000*	0.301	0.006*	0.039*
TLR-2 serum concentration [ng/mL]	8.92±3.64	8.03 (5.12-19.91)	12.31±9.03	7.97 (3.84-32.86)	17.15±9.82	18.58 (3.49-34.24)	23.98±4.67	24.98 (17.03-31.14)	0.983	0.030*	0.000*	0.088	0.005*	0.147
TLR-3 serum concentration [ng/mL]	9.95±2.70	10.15 (5.66-13.85)	10.97±7.17	9.81 (4.73-31.88)	14.35±9.50	9.90 (5.28-37.03)	14.17±8.92	8.75 (5.15-29.30)	0.589	0.426	0.860	0.117	0.417	0.132
TLR-4 serum concentration [ng/mL]	9.07±1.46	8.93 (6.30-11.26)	10.72±6.88	7.69 (5.81-30.92)	14.99±8.76	12.47 (4.29-35.61)	17.80±6.94	14.57 (9.37-30.20)	0.682	0.007*	0.000*	0.037*	0.007*	0.938
TLR-9 serum concentration [ng/mL]	9.99±3.47	10.31 (6.65-20.52)	13.15±7.85	10.36 (4.99-30.43)	17.58±8.19	19.15 (4.53-31.70)	22.66±3.70	23.13 (17.56-28.83)	0.945	0.025*	0.000*	0.095	0.007*	0.000*

*statistically significant results

Supplementary Material Table S2. Tabulated summary of the diversity of peripheral blood morphology and immunophenotype and the percentage of TLRs tested in GC patients included in the study, with particular emphasis on intestinal and diffuse type

Parameter	Intestinal type (group 1)		Diffuse type (group 2)		Control group (group 3)		p-value		
	**Mean±SD**	**Median (Range)**	**Mean±SD**	**Median (Range)**	**Mean±SD**	**Median (Range)**	1 vs. 2	1 vs. 3	2 vs.3
Age	63.38±10.74	63.00 (41.00-81.00)	61.34±9.64	63.00 (40.00-77.00)	62.28±9.50	61.00 (45.00-78.00)	0.413	0.547	0.891
White blood cells [10^3/mm^3]	6.96±0.88	6.74 (5.39-9.57)	8.22±1.10	8.22 (5.99-10.20)	7.30±0.50	7.27 (6.52-8.49)	0.000*	0.044*	0.000*

Neutrophils [10³/mm³]	3.82±0.87	3.75 (2.47-5.84)	5.03±0.90	5.13 (2.98-6.10)	4.21±0.86	4.44 (2.20-5.87)	0.000*	0.103	0.001*
Monocytes [10³/mm³]	0.41±0.11	0.37 (0.28-0.70)	0.50±0.10	0.51 (0.22-0.68)	0.45±0.12	0.47 (0.10-0.75)	0.001*	0.086	0.047*
Lymphocytes [10³/mm³]	1.99±0.44	1.93 (1.31-3.06)	2.32±0.42	2.31 (1.50-3.14)	2.57±0.56	2.61 (1.57-4.10)	0.004*	0.000*	0.080
T lymphocytes CD3+ [%]	68.37±5.04	68.46 (58.89-79.40)	70.73±4.76	72.55 (58.89-79.79)	71.59±2.68	71.28 (65.34-76.70)	0.038*	0.015*	0.959
B lymphocytes CD19+ [%]	10.81±2.42	10.45 (7.31-16.30)	10.91±.31	10.95 (7.58-16.14)	11.96±2.33	11.75 (7.62-16.82)	0.853	0.089	0.119
T lymphocytes CD3+CD4+ [%]	37.37±2.81	37.31 (31.76-42.17)	39.28±3.58	39.08 (33.11-47.08)	40.52±3.13	40.44 (34.97-46.35)	0.053	0.001*	0.172
T lymphocytes CD3+CD8+ [%]	29.43±3.84	29.75 (19.83-36.44)	26.21±3.62	25.92 (21.39-34.25)	30.30±3.08	30.49 (24.19-35.78)	0.002*	0.379	0.000*
T lymphocytes ratio CD3+CD4+/T CD3+CD8+	1.23±0.19	1.21 (0.96-1.85)	1.49±0.24	1.46 (1.05-2.02)	1.36±0.23	1.28 (1.03-1.90)	0.000*	0.021*	0.028*
T lymphocytes CD4+TLR-2+ [%]	3.75±.77	2.16 (0.89-19.05)	5.95±4.29	4.26 (1.08-17.32)	0.95±0.66	0.81 (0.13-2.70)	0.001*	0.000*	0.000*
T lymphocytes CD8+TLR-2+ [%]	3.99±6.01	2.02 (0.53-21.41)	6.17±5.08	4.44 (0.69-20.10)	1.30±1.13	0.71 (0.24-4.24)	0.001*	0.010*	0.000*
B lymphocytes CD19+TLR-2+ [%]	4.42±3.81	3.15 (1.00-15.05)	5.95±3.16	5.48 (1.52-14.40)	2.55±0.93	2.48 (0.61-4.12)	0.001*	0.034*	0.000*
T lymphocytes CD4+TLR-3+ [%]	5.25±3.87	3.54 (1.86-16.74)	9.64±4.49	11.56 (2.01-17.28)	0.64±0.33	0.60 (0.09-1.35)	0.001*	0.000*	0.000*
T lymphocytes CD8+TLR-3+ [%]	5.49±3.99	3.87 (2.25-17.12)	8.96±3.75	8.72 (3.17-17.01)	0.85±0.41	0.90 (0.07-1.76)	0.000*	0.000*	0.000*
B lymphocytes CD19+TLR-3+ [%]	7.77±6.06	5.62 (3.64-29.72)	13.25±6.96	13.02 (4.20-33.12)	1.21±0.64	0.95 (0.43-2.80)	0.000*	0.000*	0.000*
T lymphocytes CD4+TLR-4+ [%]	4.34±5.29	2.59 (1.07-21.34)	6.70±4.77	4.77 (1.30-19.40)	1.02±0.71	0.85 (0.14-2.94)	0.001*	0.005*	0.000*
T lymphocytes CD8+TLR-4+ [%]	4.58±6.69	2.42 (0.64-23.98)	6.94±5.67	4.97 (0.83-22.51)	1.40±1.22	0.77 (0.26-4.62)	0.002*	0.002*	0.000*
B lymphocytes CD19+TLR-4+ [%]	5.15±4.21	3.78 (1.20-16.86)	6.71±3.49	6.14 (1.82-16.13)	2.73±0.98	2.70 (0.66-4.24)	0.001*	0.000*	0.000*
T lymphocytes CD4+TLR-9+ [%]	6.31±3.71	4.65 (2.45-14.95)	11.01± 4.68	12.62 (2.65-19.86)	1.02±0.54	0.99 (0.19-2.28)	0.001*	0.000*	0.000*
T lymphocytes CD8+TLR-9+ [%]	6.60±3.73	5.09 (2.95-16.72)	10.27±.67	10.02 (4.17-16.69)	1.35±0.57	1.44 (0.15-2.31)	0.000*	0.000*	0.000*

B lymphocytes CD19+TLR-9+ [%]	9.24±5.08	7.40 (4.79-26.54)	15.09±6.75	14.96 (5.52-29.57)	1.84±0.68	1.77 (0.94-3.68)	0.000*	0.000*	0.000*
TLR-2 serum concentration [ng/mL]	10.28±8.35	7.92 (3.49-34.24)	19.22±7.55	21.28 (3.84-32.86)	1.32±0.44	1.21 (0.62-2.27)	0.000*	0.000*	0.000*
TLR-3 serum concentration [ng/mL]	12.70±7.94	10.99 (5.28-37.03)	11.64±7.53	7.88 (4.59-30.92)	1.15±0.51	1.03 (0.54-2.29)	0.370	0.000*	0.000*
TLR-4 serum concentration [ng/mL]	11.32±8.09	8.93 (4.29-35.61)	14.23±6.49	12.47 (4.73-31.88)	1.10±0.49	0.99 (0.52-2.21)	0.000*	0.000*	0.000*
TLR-9 serum concentration [ng/mL]	11.78±7.11	10.28 (4.53-31.70)	19.22±6.15	20.52 (4.99-30.43)	2.99±0.58	3.08 (2.03-3.92)	0.000*	0.000*	0.000*

*statistically significant results

Supplementary Material Table S3. Tabular summary of the diversity of peripheral blood morphology and immunophenotype and the percentage of TLRs tested in GC patients included in the study, with particular emphasis on gender differences

Parameter	Male with GC		Female with GC		p-value
	Mean±SD	Median (Range)	Mean±SD	Median (Range)	
Age	61.94±10.21	63.00 (40.00-77.00)	63.00±10.30	62.00 (41.00-81.00)	0.912
White blood cells [10^3/mm^3]	7.64±1.15	7.55 (5.39-10.12)	7.52±1.21	7.40 (5.79-10.20)	0.670
Neutrophils [10^3/mm^3]	4.48±1.11	5.05 (2.47-6.09)	4.36±1.00	4.12 (2.62-6.10)	0.789
Monocytes [10^3/mm^3]	0.45±0.10	0.46 (0.28-0.67)	0.47±0.13	0.41 (0.22-0.70)	0.614
Lymphocytes [10^3/mm^3]	2.12±0.44	2.20 (1.31-3.10)	2.22±0.48	2.04 (1.50-3.14)	0.570
T lymphocytes CD3+ [%]	69.36±5.15	69.77 (58.89-79.79)	69.83±4.86	71.57 (59.45-75.30)	0.477
B lymphocytes CD19+ [%]	11.33±2.36	11.17 (7.58-16.30)	10.16±2.19	9.72 (7.31-16.14)	0.065
T lymphocytes CD3+CD4+ [%]	38.40±3.57	38.06 (31.76-47.08)	38.21±3.00	37.31 (32.16-45.43)	0.801
T lymphocytes CD3+CD8+ [%]	27.54±3.82	27.46 (21.39-36.44)	28.25±4.38	29.31 (19.83-34.87)	0.570
T lymphocytes ratio CD3+CD4+/T CD3+CD8+	1.37±0.23	1.36 (0.96-2.02)	1.34±0.28	1.27 (1.04-1.99)	0.385

T lymphocytes CD4+TLR-2+ [%]	5.01±4.66	3.60 (0.89-19.05)	4.60±4.65	2.62 (0.99-17.32)	0.559
T lymphocytes CD8+TLR-2+ [%]	5.09±.33	3.50 (0.53-21.41)	5.06±6.15	2.36 (0.58-20.10)	0.507
B lymphocytes CD19+TLR-2+ [%]	5.23±3.38	4.52 (1.85-15.05)	5.11±3.87	3.82 (1.00-14.40)	0.538
T lymphocytes CD4+TLR-3+ [%]	7.98±4.78	6.15 (1.86-17.28)	6.64±4.55	6.27 (1.89-16.50)	0.297
T lymphocytes CD8+TLR-3+ [%]	7.66±4.20	5.88 (3.17-17.12)	6.57±4.21	5.21 (2.25-14.55)	0.217
B lymphocytes CD19+TLR-3+ [%]	11.42±7.69	8.33 (4.27-33.12)	9.13±5.77	6.76 (3.64-24.53)	0.458
T lymphocytes CD4+TLR-4+ [%]	5.68±5.18	4.03 (1.07-21.34)	5.27±5.15	3.14 (1.19-19.40)	0.570
T lymphocytes CD8+TLR-4+ [%]	5.76±5.94	3.92 (0.64-23.98)	5.76±6.84	2.83 (0.70-22.51)	0.518
B lymphocytes CD19+TLR-4+ [%]	5.96±3.72	5.06 (2.22-16.86)	5.89±4.26	4.58 (1.20-16.13)	0.592
T lymphocytes CD4+TLR-9+ [%]	9.16±4.83	7.51 (2.45-19.86)	7.90±4.75	8.25 (2.49-15.53)	0.368
T lymphocytes CD8+TLR-9+ [%]	8.82±3.90	7.63 (4.17-15.48)	7.86±4.40	6.86 (2.95-16.72)	0.275
B lymphocytes CD19+TLR-9+ [%]	13.00±7.11	10.96 (5.62-29.57)	10.89±5.64	8.90 (4.79-27.52)	0.477
TLR-2 serum concentration [ng/mL]	15.61±8.90	16.34 (3.49-34.24)	13.44±9.32	9.02 (3.84-32.86)	0.403
TLR-3 serum concentration [ng/mL]	11.78±7.84	8.71 (4.59-37.03)	12.65±7.80	9.88 (4.73-31.88)	0.528
TLR-4 serum concentration [ng/mL]	12.86±7.25	11.70 (4.29-35.61)	12.77±7.59	9.78 (5.66-30.92)	0.368
TLR-9 serum concentration [ng/mL]	16.15±7.47	16.85 (4.53-31.70)	14.51±7.72	11.72 (4.99-30.43)	0.412

Supplementary Material Table S4. Tabular summary of the diversity of peripheral blood morphology and immunophenotype and the percentage of tested TLRs and their soluble forms in serum in men with GC included in the study, with particular emphasis on differences in individual types of GC.

Parameter	Intestinal type		Diffuse type		p-value
	Mean±SD	Median (Range)	Mean±SD	Median (Range)	
Age	62.57±9.64	66.00 (43.00-73.00)	61.52±10.55	63.00 (40.00-77.00)	0.778
White blood cells [$10^3/mm^3$]	6.76±0.77	6.60 (5.39-8.39)	8.22±0.98	8.22 (5.99-10.12)	0.000*
Neutrophils [$10^3/mm^3$]	3.53±0.87	3.36 (2.47-5.67)	5.11±0.75	5.13 (3.57-6.09)	0.000*
Monocytes [$10^3/mm^3$]	0.37±0.09	0.34 (0.28-0.62)	0.50±0.07	0.50 (0.35-0.67)	0.000*
Lymphocytes [$10^3/mm^3$]	1.82±0.37	1.74 (1.31-2.61)	2.31±0.36	2.31 (1.57-3.10)	0.002*
T lymphocytes CD3+ [%]	68.16±5.33	68.35 (58.89-79.40)	70.16±4.87	72.23 (58.89-79.79)	0.186
B lymphocytes CD19+ [%]	11.58±2.53	11.60 (7.94-16.30)	11.16±2.23	11.03 (7.58-15.25)	0.654
T lymphocytes CD3+CD4+ [%]	37.18±2.85	37.46 (31.76-42.17)	39.22±3.77	39.08 (33.11-47.08)	0.175
T lymphocytes CD3+CD8+ [%]	29.87±3.42	29.88 (24.07-36.44)	25.99±3.25	26.16 (21.39-32.89)	0.004*
T lymphocytes ratio CD3+CD4+/T CD3+CD8+	1.20±0.13	1.21 (0.96-1.41)	1.49±0.21	1.46 (1.16-2.02)	0.000*
T lymphocytes CD4+TLR-2+ [%]	4.06±5.72	1.86 (0.89-19.05)	5.65±3.67	4.26 (1.35-15.30)	0.006*
T lymphocytes CD8+TLR-2+ [%]	4.12±6.61	1.51 (0.53-21.41)	5.74±4.15	4.44 (0.86-19.03)	0.006*
B lymphocytes CD19+TLR-2+ [%]	4.40±4.05	2.95 (1.85-15.05)	5.78±2.71	5.48 (1.99-14.19)	0.007*
T lymphocytes CD4+TLR-3+ [%]	5.52±4.43	3.76 (1.86-16.74)	9.61±4.27	10.94 (2.01-17.28)	0.020*
T lymphocytes CD8+TLR-3+ [%]	5.77±4.32	3.86 (3.27-17.12)	8.91±3.61	8.72 (3.17-17.01)	0.004*
B lymphocytes CD19+TLR-3+ [%]	8.65±8.00	5.32 (4.27-29.72)	13.27±6.87	13.02 (4.38-33.12)	0.008*
T lymphocytes CD4+TLR-4+ [%]	4.67±6.36	2.24 (1.07-21.34)	6.36±4.08	4.77 (1.62-17.14)	0.006*
T lymphocytes CD8+TLR-4+ [%]	4.72±7.37	1.81	6.45±4.63	4.97	0.006*

		(0.64-23.98)		(1.03-21.31)	
B lymphocytes CD19+TLR-4+ [%]	5.12±4.47	3.53<br>(2.22-16.86)	6.52±2.99	6.14<br>(2.39-15.89)	0.007*
T lymphocytes CD4+TLR-9+ [%]	6.30±3.66	4.94<br>(2.45-14.95)	11.06±4.57	12.58<br>(2.65-19.86)	0.009*
T lymphocytes CD8+TLR-9+ [%]	6.61±3.34	5.08<br>(4.31-15.29)	10.28±3.54	10.02<br>(4.17-15.48)	0.003*
B lymphocytes CD19+TLR-9+ [%]	9.69±6.39	7.01<br>(5.62-26.54)	15.21±6.70	14.96<br>(5.76-29.57)	0.006*
TLR-2 serum concentration [ng/mL]	10.42±9.22	8.03<br>(3.49-34.24)	19.07±6.74	21.28<br>(4.04-31.14)	0.003*
TLR-3 serum concentration [ng/mL]	13.77±9.26	12.15<br>(5.28-37.03)	10.45±6.39	7.88<br>(4.59-29.30)	0.309
TLR-4 serum concentration [ng/mL]	11.94±9.26	9.88<br>(4.29-35.61)	13.47±5.44	12.30<br>(4.97-30.20)	0.022*
TLR-9 serum concentration [ng/mL]	11.81±7.92	10.43<br>(4.53-31.70)	19.05±5.50	19.87<br>(5.25-28.83)	0.003*

*statistically significant results

Supplementary Material Table S5. Tabular summary of the diversity of peripheral blood morphology and immunophenotype and the percentage of tested TLRs and their soluble forms in serum in females with GC included in the study, with particular emphasis on differences in individual types of GC.

Parameter	Intestinal type		Diffuse type		p-value
	Mean±SD	Median (Range)	Mean±SD	Median (Range)	
Age	64.13±11.63	63.00<br>(41.00-81.00)	60.88±6.68	61.00<br>(52.00-74.00)	0.506
White blood cells [10^3/mm^3]	7.15±0.94	6.83<br>(5.79-9.57)	8.20±1.36	8.37<br>(6.30-10.20)	0.149
Neutrophils [10^3/mm^3]	4.10±0.78	4.03<br>(2.62-5.84)	4.84±1.18	5.34<br>(2.98-6.10)	0.190
Monocytes [10^3/mm^3]	0.44±0.11	0.39<br>(0.33-0.70)	0.51±0.15	0.57<br>(0.22-0.68)	0.325
Lymphocytes [10^3/mm^3]	2.15±0.43	1.98<br>(1.72-3.06)	2.36±0.54	2.40<br>(1.50-3.14)	0.392
T lymphocytes CD3+ [%]	68.56±4.74	69.30<br>(59.45-75.30)	72.22±4.12	73.82<br>(61.82-74.84)	0.056
B lymphocytes CD19+ [%]	10.10±2.07	9.84<br>(7.31-14.27)	10.26±2.40	9.37<br>(8.25-16.14)	0.925
T lymphocytes CD3+CD4+ [%]	37.55±2.76	37.10<br>(32.16-42.10)	39.45±3.05	38.69<br>(35.90-45.43)	0.238
T lymphocytes CD3+CD8+ [%]	29.02±4.16	29.75	26.79±4.41	24.28	0.294

		(19.83-34.87)		(22.66-34.25)	
T lymphocytes ratio CD3+CD4+/T CD3+CD8+	1.26±0.22	1.19<br>(1.04-1.85)	1.50±0.31	1.47<br>(1.05-1.99)	0.076
T lymphocytes CD4+TLR-2+ [%]	3.47±3.63	2.41<br>(0.99-13.30)	6.73±5.52	4.56<br>(1.08-17.32)	0.213
T lymphocytes CD8+TLR-2+ [%]	3.87±5.38	2.19<br>(0.58-18.43)	7.29±6.84	4.85<br>(0.69-20.10)	0.213
B lymphocytes CD19+TLR-2+ [%]	4.44±3.58	3.80<br>(1.00-13.91)	6.37±4.07	5.53<br>(1.52-14.40)	0.238
T lymphocytes CD4+TLR-3+ [%]	5.00±3.25	3.40<br>(1.89-12.38)	9.72±5.03	11.72<br>(2.29-16.50)	0.047*
T lymphocytes CD8+TLR-3+ [%]	5.24±3.63	4.21<br>(2.25-14.55)	9.06±4.10	9.15<br>(3.77-14.52)	0.047*
B lymphocytes CD19+TLR-3+ [%]	6.95±3.14	5.76<br>(3.64-15.22)	13.21±7.19	12.26<br>(4.20-24.53)	0.034*
T lymphocytes CD4+TLR-4+ [%]	4.03±4.03	2.89<br>(1.19-14.90)	7.59±6.14	5.11<br>(1.30-19.40)	0.213
T lymphocytes CD8+TLR-4+ [%]	4.46±5.99	2.63<br>(0.70-20.64)	8.20±7.62	5.43<br>(0.83-22.51)	0.213
B lymphocytes CD19+TLR-4+ [%]	5.19±.95	4.56<br>(1.20-15.58)	7.21±4.49	6.19<br>(1.82-16.13)	0.238
T lymphocytes CD4+TLR-9+ [%]	6.32±3.76	4.48<br>(2.49-14.23)	10.87±4.98	13.47<br>(3.01-15.53)	0.047*
T lymphocytes CD8+TLR-9+ [%]	6.59±4.07	5.54<br>(2.95-16.72)	10.24±3.98	10.51<br>(4.96-16.69)	0.065
B lymphocytes CD19+TLR-9+ [%]	8.82±3.38	7.58<br>(4.79-17.49)	14.77±6.86	14.09<br>(5.52-27.52)	0.034*
TLR-2 serum concentration [ng/mL]	10.14±7.45	7.26<br>(4.40-30.06)	19.63±9.32	22.05<br>(3.84-32.86)	0.040*
TLR-3 serum concentration [ng/mL]	11.70±6.30	9.78<br>(5.66-28.28)	14.78±9.22	10.94<br>(5.81-30.92)	0.875
TLR-4 serum concentration [ng/mL]	10.74±6.77	8.25<br>(5.41-29.15)	16.23±8.33	13.68<br>(4.73-31.88)	0.028*
TLR-9 serum concentration [ng/mL]	11.76±6.26	9.43<br>(5.71-27.83)	19.67±7.56	21.47<br>(4.99-30.43)	0.040*

*statistically significant results

Supplementary Material Table S6 Tabulated summary of Spearman's rank correlations for patients diagnosed with an intestinal type of GC.

Para zmiennych	R	t(N-2)	p
CD4+TLR-9+ [%]& sTLR9	0.340	2.049	0.049*
T CD3+ [%] & CD8+TLR-9+ [%]	0.342	2.059	0.048*
Age & CD8+TLR3+	0.345	2.080	0.046*
CD3+CD4+/T CD3+CD8+ & sTLR2	0.345	2.082	0.045*
T CD3+ [%] & CD19+TLR3+	0.346	2.085	0.045*
CD4+TLR3+ & sTLR3	0.348	2.098	0.044*
CD4+TLR3+ & sTLR2	0.352	2.130	0.041*
CD3+CD4+ [%] & CD4+TLR-9+ [%]	0.354	2.142	0.040*
Monocytes & CD19+TLR-2+ [%]	0.358	2.168	0.038*
CD19+TLR4+ & sTLR4	0.358	2.172	0.037*
T CD3+ [%] & sTLR2	0.360	2.179	0.037*
CD19+TLR-2+ [%] & sTLR4	0.362	2.198	0.035*
B CD19+ [%] & sTLR4	0.366	2.226	0.033*
CD3+CD4+ [%] & CD8+TLR-2+ [%]	0.367	2.229	0.033*
CD3+CD4+ [%] & CD8+TLR4+	0.367	2.229	0.033*
Lymphocytes & CD19+TLR3+	0.369	2.248	0.032*
B CD19+ [%] & CD8+TLR3+	0.370	2.251	0.031*
Neutrophils & sTLR2	0.370	2.255	0.031*
Monocytes & CD19+TLR4+	0.371	2.258	0.031*
T CD3+ [%] & sTLR3	0.371	2.260	0.031*
Lymphocytes & sTLR2	0.372	2.270	0.030*
Monocytes & CD8+TLR3+	0.372	2.270	0.030*
White blood cells & sTLR2	0.373	2.273	0.030*
B CD19+ [%] & sTLR2	0.375	2.286	0.029*
Neutrophils & Lymphocytes .	0.375	2.291	0.029*
T CD3+ [%] & sTLR4	0.376	2.295	0.028
CD19+TLR3+ & sTLR9	0.380	2.323	0.027*
CD8+TLR-2+ [%] & sTLR4	0.383	2.346	0.025*
CD8+TLR4+ & sTLR4	0.383	2.346	0.025*
CD19+TLR-9+ [%] & sTLR2	0.385	2.359	0.025*
CD8+TLR-9+ [%] & sTLR2	0.385	2.361	0.024*
CD3+CD4+ [%] & CD4+TLR-2+ [%]	0.388	2.382	0.023*
CD3+CD4+ [%] & CD4+TLR4+	0.388	2.382	0.023*
CD8+TLR3+ & sTLR9	0.390	2.397	0.023*
CD4+TLR-2+ [%] & sTLR4	0.390	2.399	0.022*
CD4+TLR4+ & sTLR4	0.390	2.399	0.022*
Stage & CD4+TLR-9+ [%]	0.390	2.204	0.036*
Stage & CD4+TLR3+	0.390	2.204	0.036*
CD3+CD4+/T CD3+CD8+ & CD8+TLR3+	0.397	2.448	0.020*
Age & CD8+TLR-9+ [%]	0.398	2.452	0.020*
CD8+TLR-9+ [%] & Age	0.398	2.452	0.020*
CD3+CD4+ [%] & CD19+TLR-2+ [%]	0.398	2.453	0.020*
CD8+TLR-9+ [%] & sTLR9	0.399	2.463	0.019*
CD3+CD4+/T CD3+CD8+ & sTLR9	0.399	2.466	0.019*
Monocytes & sTLR3	0.401	2.473	0.019*
Monocytes & sTLR2	0.401	2.478	0.019*
CD3+CD4+ [%] & CD19+TLR3+	0.404	2.501	0.018*
CD3+CD4+ [%] & sTLR3	0.406	2.510	0.017*
Monocytes & sTLR4	0.407	2.518	0.017*
Age & T CD3+ [%]	0.407	2.518	0.017*
T CD3+ [%] & Age	0.407	2.518	0.017*
CD3+CD4+/T CD3+CD8+ & CD19+TLR-9+ [%]	0.407	2.523	0.017*
CD3+CD4+ [%] & CD19+TLR4+	0.408	2.527	0.017*
Lymphocytes & sTLR3	0.410	2.539	0.016*
Monocytes & T lymphocytes CD3+CD4+ [%]	0.410	2.540	0.016*
CD19+TLR-9+ [%] & sTLR9	0.412	2.555	0.016*
Neutrophils & CD8+TLR3+	0.413	2.564	0.015*
Neutrophils & T CD3+ [%]	0.415	2.577	0.015*
T CD3+ [%] & sTLR9	0.419	2.607	0.014*
White blood cells & sTLR9	0.420	2.620	0.013*
Neutrophils & CD3+CD4+/T CD3+CD8+	0.421	2.628	0.013*
Neutrophils & sTLR9	0.421	2.628	0.013*
CD4+TLR-2+ [%] & CD8+TLR-9+ [%]	0.422	2.633	0.013*

CD8+TLR-9+ [%] & CD4+TLR4+	0.422	2.633	0.013*
Monocytes & CD8+TLR-9+ [%]	0.423	2.644	0.013*
CD8+TLR3+ & sTLR2	0.424	2.649	0.012*
Lymphocytes & sTLR4	0.424	2.651	0.012*
CD19+TLR3+ & sTLR2	0.425	2.656	0.012*
Monocytes & CD4+TLR3+	0.425	2.660	0.012*
CD8+TLR-2+ [%] & CD8+TLR-9+ [%]	0.426	2.661	0.012*
CD8+TLR-9+ [%] & CD8+TLR4+	0.426	2.661	0.012*
CD3+CD4+ [%] & CD3+CD8+ [%]	0.426	2.662	0.012*
Age & CD3+CD8+ [%]	0.429	2.685	0.011*
CD3+CD8+ [%] & Age	0.429	2.685	0.011*
B CD19+ [%] & sTLR9	0.430	2.696	0.011*
CD19+TLR-2+ [%] & CD8+TLR-9+ [%]	0.431	2.702	0.011*
CD8+TLR-9+ [%] & CD19+TLR4+	0.431	2.704	0.011*
Lymphocytes & sTLR9	0.432	2.709	0.011*
CD3+CD4+/T CD3+CD8+ & CD8+TLR-2+ [%]	0.435	2.731	0.010*
CD3+CD4+/T CD3+CD8+ & CD8+TLR4+	0.435	2.731	0.010*
Neutrophils & CD4+TLR3+	0.441	2.780	0.009*
T CD3+ [%] & CD19+TLR-9+ [%]	0.442	2.786	0.009*
CD3+CD4+/T CD3+CD8+ & CD4+TLR-2+ [%]	0.442	2.788	0.009*
CD3+CD4+/T CD3+CD8+ & CD4+TLR4+	0.442	2.788	0.009*
CD3+CD4+/T CD3+CD8+ & CD19+TLR-2+ [%]	0.448	2.835	0.008*
CD19+TLR-2+ [%] & CD4+TLR-9+ [%]	0.449	2.846	0.008*
Neutrophils & T lymphocytes CD3+CD4+ [%]	0.450	2.854	0.008*
Monocytes & CD4+TLR-9+ [%]	0.450	2.854	0.008*
Stage & CD19+TLR-9+ [%]	0.451	2.624	0.014*
Stage & CD19+TLR3+	0.451	2.624	0.014*
CD4+TLR-2+ [%] & CD4+TLR-9+ [%]	0.451	2.857	0.007*
CD4+TLR-9+ [%]& CD4+TLR4+	0.451	2.857	0.007*
Monocytes & sTLR9	0.451	2.862	0.007*
Neutrophils & CD8+TLR-9+ [%]	0.452	2.864	0.007*
CD8+TLR-9+ [%] & Neutrophils .	0.452	2.864	0.007*
CD8+TLR-2+ [%] & CD4+TLR-9+ [%]	0.453	2.876	0.007*

CD4+TLR-9+ [%]& CD8+TLR4+	0.453	2.876	0.007*
CD4+TLR-9+ [%]& CD19+TLR4+	0.454	2.885	0.007*
Lymphocytes & CD19+TLR-9+ [%]	0.456	2.900	0.007*
CD3+CD4+ [%] & sTLR4	0.459	2.923	0.006*
Lymphocytes & CD3+CD8+ [%]	0.460	2.934	0.006*
CD3+CD4+/T CD3+CD8+ & CD19+TLR4+	0.461	2.936	0.006*
B CD19+ [%] & CD8+TLR-9+ [%]	0.463	2.953	0.006*
CD4+TLR-2+ [%] & CD19+TLR-9+ [%]	0.464	2.967	0.006*
CD19+TLR-9+ [%] & CD4+TLR4+	0.464	2.967	0.006*
CD8+TLR-2+ [%] & CD19+TLR-9+ [%]	0.465	2.969	0.006*
CD19+TLR-9+ [%] & CD8+TLR4+	0.465	2.969	0.006*
White blood cells & Monocytes .	0.466	2.979	0.005*
CD19+TLR4+ & CD4+TLR3+	0.467	2.990	0.005*
CD3+CD4+ [%] & CD8+TLR3+	0.468	2.993	0.005*
CD19+TLR-2+ [%] & CD4+TLR3+	0.468	2.995	0.005*
CD3+CD4+/T CD3+CD8+ & CD8+TLR-9+ [%]	0.471	3.020	0.005*
CD19+TLR-9+ [%] & CD4+TLR3+	0.471	3.023	0.005*
Neutrophils & CD4+TLR-9+ [%]	0.476	3.065	0.004*
CD19+TLR-2+ [%] & sTLR9	0.478	3.082	0.004*
CD4+TLR-9+ [%]& CD19+TLR3+	0.478	3.082	0.004*
CD8+TLR-2+ [%] & sTLR9	0.479	3.086	0.004*
CD8+TLR4+ & sTLR9	0.479	3.086	0.004*
CD19+TLR4+ & sTLR9	0.480	3.092	0.004*
CD4+TLR-2+ [%] & sTLR9	0.482	3.108	0.004*
CD4+TLR4+ & sTLR9	0.482	3.108	0.004*
White blood cells & CD8+TLR3+	0.483	3.120	0.004*
CD19+TLR4+ & CD8+TLR3+	0.485	3.134	0.004*
CD4+TLR-2+ [%] & CD4+TLR3+	0.485	3.135	0.004*
CD4+TLR4+ & CD4+TLR3+	0.485	3.135	0.004*
CD8+TLR-2+ [%] & CD4+TLR3+	0.487	3.153	0.004*
CD8+TLR4+ & CD4+TLR3+	0.487	3.153	0.004*
CD4+TLR3+ & CD19+TLR3+	0.487	3.158	0.003*
CD19+TLR-2+ [%] & CD8+TLR3+	0.496	3.228	0.003*

CD3+CD4+ [%] & CD19+TLR-9+ [%]	0.498	3.248	0.003*
CD4+TLR-9+ [%]& CD19+TLR-9+ [%]	0.499	3.254	0.003*
CD3+CD4+ [%] & sTLR2	0.500	3.267	0.003*
CD19+TLR4+ & sTLR2	0.509	3.344	0.002*
CD19+TLR-2+ [%] & CD19+TLR-9+ [%]	0.509	3.349	0.002*
CD19+TLR-9+ [%] & CD19+TLR-2+ [%]	0.509	3.349	0.002*
CD4+TLR-2+ [%] & CD8+TLR3+	0.510	3.351	0.002*
CD4+TLR4+ & CD8+TLR3+	0.510	3.351	0.002*
CD8+TLR-2+ [%] & CD8+TLR3+	0.513	3.378	0.002*
CD8+TLR4+ & CD8+TLR3+	0.513	3.378	0.002*
CD19+TLR-9+ [%] & CD19+TLR4+	0.515	3.394	0.002*
CD3+CD4+ [%] & CD8+TLR-9+ [%]	0.517	3.418	0.002*
White blood cells & CD8+TLR-9+ [%]	0.517	3.419	0.002*
CD19+TLR-2+ [%] & sTLR2	0.519	3.437	0.002*
B CD19+ [%] & T lymphocytes CD3+CD4+ [%]	0.521	3.449	0.002*
White blood cells & CD19+TLR3+	0.521	3.450	0.002*
White blood cells & CD4+TLR3+	0.529	3.522	0.001*
CD8+TLR-2+ [%] & sTLR2	0.542	3.652	0.001*
CD8+TLR4+ & sTLR2	0.542	3.652	0.001*
CD4+TLR-2+ [%] & sTLR2	0.545	3.676	0.001*
CD4+TLR4+ & sTLR2	0.545	3.676	0.001*
sTLR2 & CD4+TLR4+	0.545	3.676	0.001*
Monocytes & CD3+CD8+ [%]	0.553	3.755	0.001*
White blood cells & CD4+TLR-9+ [%]	0.554	3.760	0.001*
CD3+CD4+ [%] & sTLR9	0.554	3.766	0.001*
CD4+TLR-2+ [%] & CD19+TLR3+	0.560	3.824	0.001*
CD4+TLR4+ & CD19+TLR3+	0.560	3.824	0.001*
CD8+TLR-2+ [%] & CD19+TLR3+	0.560	3.827	0.001*
CD8+TLR4+ & CD19+TLR3+	0.560	3.827	0.001*
CD8+TLR-9+ [%] & CD4+TLR3+	0.562	3.839	0.001*
CD19+TLR4+ & CD19+TLR3+	0.562	3.844	0.001*
CD4+TLR-9+ [%]& CD8+TLR3+	0.567	3.896	0.000*
Lymphocytes & T CD3+ [%]	0.571	3.934	0.000*

Monocytes & CD19+TLR3+	0.572	3.945	0.000*
CD19+TLR-2+ [%] & CD19+TLR3+	0.574	3.967	0.000*
CD4+TLR3+ & CD8+TLR3+	0.577	4.002	0.000*
CD4+TLR-9+ [%]& CD8+TLR-9+ [%]	0.578	4.010	0.000*
CD8+TLR-9+ [%] & CD4+TLR-9+ [%]	0.578	4.010	0.000*
Neutrophils & CD19+TLR3+	0.589	4.127	0.000*
White blood cells & CD19+TLR-9+ [%]	0.594	4.181	0.000*
White blood cells & T lymphocytes CD3+CD4+ [%]	0.602	4.269	0.000*
Stage & CD8+TLR-9+ [%]	0.623	4.135	0.000*
Stage & CD8+TLR3+	0.623	4.135	0.000*
Neutrophils & CD8+TLR-2+ [%]	0.645	4.776	0.000*
Neutrophils & CD8+TLR4+	0.645	4.776	0.000*
White blood cells & CD3+CD4+/T CD3+CD8+	0.646	4.786	0.000*
Monocytes & CD19+TLR-9+ [%]	0.647	4.799	0.000*
Neutrophils & CD4+TLR-2+ [%]	0.651	4.854	0.000*
Neutrophils & CD4+TLR4+	0.651	4.854	0.000*
Monocytes & T CD3+ [%]	0.653	4.875	0.000*
Neutrophils & Monocytes .	0.658	4.939	0.000*
Neutrophils & CD19+TLR-9+ [%]	0.661	4.983	0.000*
B CD19+ [%] & CD3+CD4+/T CD3+CD8+	0.672	5.138	0.000*
T CD3+ [%] & T lymphocytes CD3+CD4+ [%]	0.702	5.571	0.000*
Neutrophils & CD19+TLR-2+ [%]	0.740	6.219	0.000*
Neutrophils & CD19+TLR4+	0.757	6.557	0.000*
CD3+CD4+ [%] & CD3+CD4+/T CD3+CD8+	0.763	6.685	0.000*
White blood cells & Neutrophils .	0.766	6.730	0.000*
Monocytes & Lymphocytes .	0.766	6.747	0.000*
sTLR2 & sTLR3	0.780	7.050	0.000*
White blood cells & CD8+TLR-2+ [%]	0.788	7.242	0.000*
White blood cells & CD8+TLR4+	0.788	7.242	0.000*
CD8+TLR-9+ [%] & CD19+TLR3+	0.795	7.422	0.000*
CD4+TLR-2+ [%] & White blood cells .	0.797	7.452	0.000*
White blood cells & CD4+TLR4+	0.797	7.452	0.000*
CD19+TLR-9+ [%] & CD8+TLR3+	0.799	7.522	0.000*

sTLR3 & sTLR9	0.807	7.718	0.000*
White blood cells & CD19+TLR-2+ [%]	0.826	8.274	0.000*
CD8+TLR-9+ [%] & CD19+TLR-9+ [%]	0.831	8.454	0.000*
CD8+TLR3+ & CD19+TLR3+	0.836	8.626	0.000*
White blood cells & CD19+TLR4+	0.844	8.919	0.000*
T CD3+ [%] & CD3+CD8+ [%]	0.875	10.207	0.000*
sTLR2 & sTLR4	0.912	12.605	0.000*
sTLR4 & sTLR9	0.922	13.423	0.000*
CD19+TLR-9+ [%] & CD19+TLR3+	0.946	16.490	0.000*
CD8+TLR-2+ [%] & CD19+TLR4+	0.956	18.515	0.000*
CD8+TLR4+ & CD19+TLR4+	0.956	18.515	0.000*
CD4+TLR-2+ [%] & CD19+TLR4+	0.957	18.725	0.000*
CD4+TLR4+ & CD19+TLR4+	0.957	18.725	0.000*

sTLR4 & sTLR3	0.960	19.461	0.000*
CD8+TLR-2+ [%] & CD19+TLR-2+ [%]	0.964	20.417	0.000*
CD19+TLR-2+ [%] & CD8+TLR4+	0.964	20.417	0.000*
CD19+TLR-2+ [%] & CD4+TLR-2+ [%]	0.965	20.741	0.000*
CD8+TLR-9+ [%] & CD8+TLR3+	0.972	23.512	0.000*
sTLR2 & sTLR9	0.975	24.944	0.000*
CD4+TLR-9+ [%]& CD4+TLR3+	0.992	45.491	0.000*
CD19+TLR-2+ [%] & CD19+TLR4+	0.998	93.267	0.000*
CD4+TLR-2+ [%] & CD8+TLR-2+ [%]	0.998	97.418	0.000*
CD4+TLR-2+ [%] & CD8+TLR4+	0.998	97.418	0.000*
CD8+TLR-2+ [%] & CD4+TLR4+	0.998	97.418	0.000*
CD4+TLR4+ & CD8+TLR4+	0.998	97.418	0.000*

*statistically significant results

Supplementary Material Table S7 Tabulated summary of Spearman's rank correlations for patients diagnosed with a diffuse type of GC.

Para zmiennych	R	t(N-2)	p
CD3+CD8+ [%] & CD8+TLR-2+ [%]	-0.501	-3.274	0.003*
CD8+TLR-2+ [%] & CD3+CD8+ [%]	-0.501	-3.274	0.003*
CD3+CD8+ [%] & CD8+TLR-4+	-0.501	-3.274	0.003*
CD3+CD8+ [%] & CD4+TLR-2+ [%]	-0.497	-3.241	0.003*
CD4+TLR-2+ [%] & CD3+CD8+ [%]	-0.497	-3.241	0.003*
CD3+CD8+ [%] & CD4+TLR-4+	-0.490	-3.176	0.003*
CD3+CD8+ [%] & CD19+TLR-2+ [%]	-0.381	-2.330	0.026*
CD19+TLR-2+ [%] & CD3+CD8+ [%]	-0.381	-2.330	0.026*
CD3+CD8+ [%] & CD19+TLR-4+	-0.355	-2.147	0.039*
CD3+CD8+ [%] & CD4+TLR-3+	-0.348	-2.097	0.044*
CD3+CD8+ [%] & CD4+TLR-9+ [%]	-0.342	-2.058	0.048*
CD4+TLR-9+ [%] & CD3+CD8+ [%]	-0.342	-2.058	0.048*
Age & Monocytes	0.353	2.134	0.041*
sTLR-3 & sTLR-9 [ng/mL]	0.358	2.172	0.037*

Age & CD19+TLR-9+ [%]	0.360	2.185	0.036*
CD3+CD4+ [%] & CD8+TLR-2+ [%]	0.363	2.201	0.035*
CD3+CD4+ [%] & CD8+TLR-4+	0.363	2.201	0.035*
Stage (TNM) [A-C] & sTLR-3	0.374	2.096	0.046*
CD3+CD4+ [%] & CD4+TLR-2+ [%]	0.376	2.294	0.029*
sTLR-2 & sTLR-3	0.377	2.303	0.028*
Age & CD8+TLR-3+	0.379	2.315	0.027*
CD19+ [%] & CD3+CD8+ [%]	0.381	2.329	0.026*
CD3+CD4+ [%] & CD4+TLR-4+	0.382	2.337	0.026*
Neutrophils & sTLR-3	0.387	2.371	0.024*
Stage (TNM) [A-C] & sTLR-2	0.387	2.181	0.038*
CD19+TLR-3+ & sTLR-3	0.389	2.386	0.023*
CD19+TLR-9+ [%] & sTLR-3	0.389	2.389	0.023*
White blood cells & sTLR-3	0.390	2.398	0.023*
CD3+ [%] & CD8+TLR-9+ [%]	0.391	2.404	0.022*

CD3+ [%] & CD19+ [%]	0.393	2.417	0.022*
Age & ratio CD3+CD4+/T CD3+CD8+	0.397	2.449	0.020*
ratio CD3+CD4+/T CD3+CD8+ & Age	0.397	2.449	0.020*
CD3+CD4+ [%] & sTLR-3	0.408	2.525	0.017*
CD3+ [%] & CD19+TLR-2+ [%]	0.411	2.547	0.016*
CD3+CD4+ [%] & sTLR-2	0.416	2.584	0.015*
Age & CD8+TLR-9+ [%]	0.416	2.587	0.014*
CD8+TLR-9+ [%] & Age	0.416	2.587	0.014*
Lymphocytes & sTLR-3	0.417	2.593	0.014*
CD3+ [%] & CD3+CD8+ [%]	0.420	2.616	0.013*
Age & CD3+CD4+ [%]	0.422	2.634	0.013*
CD3+ [%] & CD8+TLR-3+	0.423	2.639	0.013*
CD3+ [%] & CD19+TLR-4+	0.428	2.679	0.012*
CD3+ [%] & ratio CD3+CD4+/T CD3+CD8+	0.435	2.736	0.010*
ratio CD3+CD4+/T CD3+CD8+ & CD3+ [%]	0.435	2.736	0.010*
Monocytes & sTLR-3	0.445	2.813	0.008*
CD4+TLR-9+ [%] & sTLR-4	0.446	2.821	0.008*
CD3+CD4+ [%] & sTLR-9 [ng/mL]	0.449	2.839	0.008*
CD3+ [%] & CD19+TLR-3+	0.451	2.857	0.007*
CD3+ [%] & CD19+TLR-9+ [%]	0.458	2.915	0.006*
CD3+ [%] & sTLR-2	0.459	2.921	0.006*
CD3+CD4+ [%] & sTLR-4	0.460	2.932	0.006*
CD3+ [%] & sTLR-9 [ng/mL]	0.470	3.014	0.005*
Lymphocytes & CD3+ [%]	0.480	3.095	0.004*
Monocytes & CD3+CD4+ [%]	0.486	3.148	0.004*
CD3+CD4+ [%] & CD19+TLR-2+ [%]	0.493	3.208	0.003*
CD3+ [%] & CD3+CD4+ [%]	0.497	3.240	0.003*
Lymphocytes & CD3+CD4+ [%]	0.502	3.286	0.002*
CD3+CD4+ [%] & CD19+TLR-4+	0.505	3.306	0.002*
CD4+TLR-3+ & sTLR-4	0.516	3.410	0.002*
CD3+CD4+ [%] & CD19+TLR-3+	0.519	3.434	0.002*
Neutrophils & CD3+ [%]	0.526	3.501	0.001*
CD3+CD4+ [%] & CD19+TLR-9+ [%]	0.531	3.544	0.001*

Stage (TNM) [A-C] & sTLR-4	0.534	3.278	0.003*
Neutrophils & CD3+CD4+ [%]	0.549	3.711	0.001*
White blood cells & CD3+ [%]	0.561	3.830	0.001*
Monocytes & CD3+ [%]	0.567	3.897	0.000*
CD3+CD4+ [%] & CD8+TLR-3+	0.570	3.922	0.000*
CD3+CD4+ [%] & CD8+TLR-9+ [%]	0.572	3.946	0.000*
White blood cells & CD3+CD4+ [%]	0.602	4.260	0.000*
CD3+ [%] & sTLR-4	0.633	4.630	0.000*
ratio CD3+CD4+/T CD3+CD8+ & CD4+TLR-9+ [%]	0.640	4.709	0.000*
CD4+TLR-9+ [%] & CD19+TLR-9+ [%]	0.644	4.764	0.000*
CD3+ [%] & sTLR-3	0.646	4.785	0.000*
CD4+TLR-9+ [%] & CD19+TLR-3+	0.656	4.914	0.000*
CD8+TLR-9+ [%] & sTLR-4	0.662	5.003	0.000*
ratio CD3+CD4+/T CD3+CD8+ & CD4+TLR-3+	0.667	5.066	0.000*
ratio CD3+CD4+/T CD3+CD8+ & sTLR-4	0.678	5.218	0.000*
Monocytes & CD4+TLR-9+ [%]	0.689	5.372	0.000*
CD8+TLR-3+ & sTLR-4	0.690	5.395	0.000*
CD19+TLR-9+ [%] & CD4+TLR-3+	0.699	5.526	0.000*
CD8+TLR-2+ [%] & sTLR-4	0.700	5.538	0.000*
CD8+TLR-4+ & sTLR-4	0.700	5.538	0.000*
CD4+TLR-2+ [%] & sTLR-4	0.701	5.558	0.000*
CD4+TLR-4+ & sTLR-4	0.705	5.628	0.000*
Monocytes & sTLR-4	0.707	5.654	0.000*
CD4+TLR-9+ [%] & CD8+TLR-9+ [%]	0.709	5.687	0.000*
CD4+TLR-3+ & CD19+TLR-3+	0.716	5.803	0.000*
CD19+TLR-9+ [%] & sTLR-4	0.718	5.834	0.000*
CD19+TLR-3+ & sTLR-4	0.720	5.862	0.000*
CD19+TLR-2+ [%] & sTLR-4	0.723	5.912	0.000*
CD19+TLR-4+ & sTLR-4	0.727	5.983	0.000*
CD4+TLR-9+ [%] & CD8+TLR-3+	0.730	6.050	0.000*
White blood cells & CD4+TLR-9+ [%]	0.736	6.154	0.000*
Monocytes & CD4+TLR-3+	0.739	6.213	0.000*
CD8+TLR-9+ [%] & CD4+TLR-3+	0.741	6.242	0.000*

sTLR-4 & sTLR-3	0.747	6.356	0.000*
CD4+TLR-9+ [%] & sTLR-9 [ng/mL]	0.752	6.444	0.000*
CD8+TLR-2+ [%] & CD4+TLR-9+ [%]	0.756	6.530	0.000*
CD4+TLR-9+ [%] & CD8+TLR-4+	0.756	6.530	0.000*
CD4+TLR-9+ [%] & sTLR-2	0.762	6.658	0.000*
Monocytes & ratio CD3+CD4+/T CD3+CD8+	0.763	6.667	0.000*
CD4+TLR-2+ [%] & CD4+TLR-9+ [%]	0.766	6.745	0.000*
Neutrophils & CD4+TLR-9+ [%]	0.772	6.867	0.000*
Monocytes & CD8+TLR-9+ [%]	0.776	6.954	0.000*
CD4+TLR-9+ [%] & CD4+TLR-4+	0.776	6.963	0.000*
CD8+TLR-9+ [%] & sTLR-9 [ng/mL]	0.777	6.980	0.000*
Monocytes & CD19+TLR-3+	0.779	7.030	0.000*
CD3+CD4+ [%] & ratio CD3+CD4+/T CD3+CD8+	0.779	7.034	0.000*
Lymphocytes & CD4+TLR-9+ [%]	0.781	7.076	0.000*
Monocytes & CD19+TLR-9+ [%]	0.782	7.087	0.000*
ratio CD3+CD4+/T CD3+CD8+ & CD8+TLR-2+ [%]	0.783	7.119	0.000*
ratio CD3+CD4+/T CD3+CD8+ & CD8+TLR-4+	0.783	7.119	0.000*
White blood cells & CD4+TLR-3+	0.783	7.121	0.000*
CD8+TLR-9+ [%] & CD19+TLR-3+	0.783	7.125	0.000*
CD8+TLR-9+ [%] & CD19+TLR-9+ [%]	0.785	7.176	0.000*
White blood cells & sTLR-4	0.786	7.196	0.000*
CD4+TLR-3+ & CD8+TLR-3+	0.787	7.214	0.000*
Neutrophils & sTLR-4	0.789	7.253	0.000*
Monocytes & CD8+TLR-2+ [%]	0.794	7.381	0.000*
Monocytes & CD8+TLR-4+	0.794	7.381	0.000*
CD8+TLR-2+ [%] & sTLR-9 [ng/mL]	0.795	7.410	0.000*
CD8+TLR-4+ & sTLR-9 [ng/mL]	0.795	7.410	0.000*
sTLR-4 & sTLR-9 [ng/mL]	0.797	7.467	0.000*
ratio CD3+CD4+/T CD3+CD8+ & CD4+TLR-2+ [%]	0.798	7.492	0.000*
CD4+TLR-3+ & sTLR-9 [ng/mL]	0.800	7.530	0.000*
Monocytes & CD4+TLR-2+ [%]	0.801	7.576	0.000*
CD4+TLR-9+ [%] & CD19+TLR-4+	0.802	7.596	0.000*
CD19+TLR-2+ [%] & CD4+TLR-9+ [%]	0.802	7.600	0.000*
ratio CD3+CD4+/T CD3+CD8+ & CD4+TLR-4+	0.805	7.674	0.000*
CD4+TLR-2+ [%] & sTLR-9 [ng/mL]	0.807	7.737	0.000*
CD19+TLR-9+ [%] & CD8+TLR-3+	0.808	7.750	0.000*
CD4+TLR-3+ & sTLR-2	0.810	7.811	0.000*
Monocytes & CD4+TLR-4+	0.810	7.822	0.000*
ratio CD3+CD4+/T CD3+CD8+ & sTLR-9 [ng/mL]	0.811	7.833	0.000*
Monocytes & CD8+TLR-3+	0.813	7.887	0.000*
CD8+TLR-2+ [%] & CD4+TLR-3+	0.813	7.889	0.000*
CD8+TLR-4+ & CD4+TLR-3+	0.813	7.889	0.000*
CD4+TLR-4+ & sTLR-9 [ng/mL]	0.813	7.904	0.000*
Monocytes & sTLR-9 [ng/mL]	0.814	7.926	0.000*
CD8+TLR-3+ & sTLR-9 [ng/mL]	0.814	7.929	0.000*
CD8+TLR-3+ & CD19+TLR-3+	0.814	7.935	0.000*
Neutrophils & CD4+TLR-3+	0.818	8.036	0.000*
sTLR-2 & sTLR-4	0.818	8.045	0.000*
ratio CD3+CD4+/T CD3+CD8+ & sTLR-2	0.818	8.059	0.000*
CD4+TLR-2+ [%] & CD4+TLR-3+	0.821	8.146	0.000*
CD8+TLR-9+ [%] & sTLR-2	0.822	8.170	0.000*
ratio CD3+CD4+/T CD3+CD8+ & CD19+TLR-3+	0.825	8.243	0.000*
ratio CD3+CD4+/T CD3+CD8+ & CD19+TLR-9+ [%]	0.826	8.291	0.000*
CD19+TLR-9+ [%] & ratio CD3+CD4+/T CD3+CD8+	0.826	8.291	0.000*
White blood cells & CD8+TLR-9+ [%]	0.827	8.313	0.000*
Lymphocytes & CD4+TLR-3+	0.827	8.315	0.000*
CD8+TLR-2+ [%] & CD19+TLR-9+ [%]	0.828	8.349	0.000*
CD19+TLR-9+ [%] & CD8+TLR-4+	0.828	8.349	0.000*
CD4+TLR-4+ & CD4+TLR-3+	0.830	8.431	0.000*
Monocytes & sTLR-2	0.830	8.434	0.000*
CD4+TLR-2+ [%] & CD19+TLR-9+ [%]	0.831	8.454	0.000*
CD19+TLR-9+ [%] & sTLR-9 [ng/mL]	0.833	8.512	0.000*
Lymphocytes & CD19+TLR-9+ [%]	0.834	8.547	0.000*
Lymphocytes & sTLR-4	0.835	8.573	0.000*
CD19+TLR-9+ [%] & CD4+TLR-4+	0.837	8.655	0.000*
CD19+TLR-3+ & sTLR-9 [ng/mL]	0.840	8.775	0.000*

Lymphocytes & CD19+TLR-3+	0.841	8.791	0.000*
Neutrophils & CD19+TLR-9+ [%]	0.843	8.852	0.000*
Neutrophils & CD19+TLR-3+	0.844	8.885	0.000*
CD8+TLR-2+ [%] & CD8+TLR-9+ [%]	0.845	8.927	0.000*
CD8+TLR-9+ [%] & CD8+TLR-4+	0.845	8.927	0.000*
White blood cells & CD8+TLR-2+ [%]	0.846	8.973	0.000*
White blood cells & CD8+TLR-4+	0.846	8.973	0.000*
Monocytes & Lymphocytes	0.847	9.021	0.000*
White blood cells & Monocytes	0.849	9.086	0.000*
CD4+TLR-2+ [%] & CD8+TLR-9+ [%]	0.849	9.097	0.000*
Lymphocytes & ratio CD3+CD4+/T CD3+CD8+	0.849	9.105	0.000*
CD19+TLR-4+ & CD4+TLR-3+	0.850	9.123	0.000*
CD8+TLR-2+ [%] & CD19+TLR-3+	0.851	9.153	0.000*
CD8+TLR-4+ & CD19+TLR-3+	0.851	9.153	0.000*
Neutrophils & CD8+TLR-9+ [%]	0.851	9.156	0.000*
CD19+TLR-2+ [%] & CD4+TLR-3+	0.851	9.164	0.000*
White blood cells & CD4+TLR-2+ [%]	0.852	9.189	0.000*
CD4+TLR-2+ [%] & CD19+TLR-3+	0.853	9.256	0.000*
White blood cells & CD19+TLR-9+ [%]	0.854	9.295	0.000*
White blood cells & CD8+TLR-3+	0.854	9.295	0.000*
White blood cells & CD19+TLR-3+	0.855	9.314	0.000*
CD8+TLR-3+ & sTLR-2	0.856	9.364	0.000*
CD8+TLR-2+ [%] & sTLR-2	0.857	9.398	0.000*
CD8+TLR-4+ & sTLR-2	0.857	9.398	0.000*
CD4+TLR-4+ & CD19+TLR-3+	0.857	9.424	0.000*
CD8+TLR-9+ [%] & CD4+TLR-4+	0.859	9.484	0.000*
Neutrophils & CD8+TLR-2+ [%]	0.860	9.513	0.000*
Neutrophils & CD8+TLR-4+	0.860	9.513	0.000*
Neutrophils & ratio CD3+CD4+/T CD3+CD8+	0.860	9.543	0.000*
White blood cells & CD4+TLR-4+	0.860	9.546	0.000*
White blood cells & sTLR-9 [ng/mL]	0.861	9.592	0.000*
CD19+TLR-9+ [%] & sTLR-2	0.862	9.602	0.000*
CD19+TLR-2+ [%] & sTLR-9 [ng/mL]	0.864	9.704	0.000*

CD19+TLR-4+ & sTLR-9 [ng/mL]	0.865	9.766	0.000*
CD4+TLR-2+ [%] & sTLR-2	0.866	9.794	0.000*
Neutrophils & CD4+TLR-2+ [%]	0.866	9.808	0.000*
Lymphocytes & CD8+TLR-9+ [%]	0.866	9.815	0.000*
Neutrophils & Monocytes	0.866	9.819	0.000*
Monocytes & CD19+TLR-2+ [%]	0.867	9.860	0.000*
CD19+TLR-3+ & sTLR-2	0.870	9.967	0.000*
CD4+TLR-4+ & sTLR-2	0.872	10.058	0.000*
ratio CD3+CD4+/T CD3+CD8+ & CD8+TLR-9+ [%]	0.872	10.069	0.000*
White blood cells & ratio CD3+CD4+/T CD3+CD8+	0.872	10.098	0.000*
White blood cells & sTLR-2	0.875	10.207	0.000*
CD19+TLR-2+ [%] & CD19+TLR-9+ [%]	0.875	10.244	0.000*
Neutrophils & CD4+TLR-4+	0.876	10.253	0.000*
CD19+TLR-9+ [%] & CD19+TLR-4+	0.878	10.368	0.000*
Monocytes & CD19+TLR-4+	0.879	10.421	0.000*
ratio CD3+CD4+/T CD3+CD8+ & CD19+TLR-2+ [%]	0.879	10.424	0.000*
ratio CD3+CD4+/T CD3+CD8+ & CD8+TLR-3+	0.879	10.429	0.000*
Lymphocytes & sTLR-9 [ng/mL]	0.880	10.493	0.000*
Neutrophils & CD8+TLR-3+	0.882	10.583	0.000*
Neutrophils & sTLR-9 [ng/mL]	0.882	10.600	0.000*
CD19+TLR-2+ [%] & CD8+TLR-9+ [%]	0.882	10.603	0.000*
ratio CD3+CD4+/T CD3+CD8+ & CD19+TLR-4+	0.884	10.670	0.000*
CD8+TLR-2+ [%] & CD8+TLR-3+	0.884	10.704	0.000*
CD8+TLR-4+ & CD8+TLR-3+	0.884	10.704	0.000*
CD19+TLR-4+ & CD19+TLR-3+	0.887	10.841	0.000*
CD8+TLR-9+ [%] & CD19+TLR-4+	0.887	10.850	0.000*
CD19+TLR-2+ [%] & CD19+TLR-3+	0.887	10.894	0.000*
Lymphocytes & CD8+TLR-2+ [%]	0.895	11.334	0.000*
Lymphocytes & CD8+TLR-4+	0.895	11.334	0.000*
CD19+TLR-2+ [%] & sTLR-2	0.896	11.437	0.000*
CD4+TLR-4+ & CD8+TLR-3+	0.897	11.463	0.000*
CD19+TLR-4+ & sTLR-2	0.897	11.467	0.000*
Lymphocytes & CD8+TLR-3+	0.897	11.507	0.000*

Neutrophils & sTLR-2	0.902	11.804	0.000*
Lymphocytes & CD4+TLR-2+ [%]	0.904	11.967	0.000*
Lymphocytes & sTLR-2	0.909	12.323	0.000*
Lymphocytes & CD4+TLR-4+	0.912	12.617	0.000*
CD19+TLR-2+ [%] & CD8+TLR-3+	0.917	13.031	0.000*
CD19+TLR-4+ & CD8+TLR-3+	0.919	13.212	0.000*
White blood cells & CD19+TLR-2+ [%]	0.930	14.276	0.000*
Neutrophils & CD19+TLR-2+ [%]	0.933	14.670	0.000*
White blood cells & CD19+TLR-4+	0.935	14.859	0.000*
Neutrophils & CD19+TLR-4+	0.936	14.995	0.000*
CD8+TLR-2+ [%] & CD19+TLR-4+	0.945	16.400	0.000*
CD8+TLR-4+ & CD19+TLR-4+	0.945	16.400	0.000*
White blood cells & Lymphocytes	0.946	16.426	0.000*
Lymphocytes & CD19+TLR-2+ [%]	0.947	16.746	0.000*
Lymphocytes & CD19+TLR-4+	0.950	17.211	0.000*
CD4+TLR-2+ [%] & CD19+TLR-4+	0.954	17.981	0.000*
CD8+TLR-2+ [%] & CD19+TLR-2+ [%]	0.957	18.725	0.000*
CD19+TLR-2+ [%] & CD8+TLR-4+	0.957	18.725	0.000*
CD4+TLR-4+ & CD19+TLR-4+	0.961	19.600	0.000*
CD4+TLR-2+ [%] & CD19+TLR-2+ [%]	0.965	20.788	0.000*
CD4+TLR-9+ [%] & CD4+TLR-3+	0.968	21.703	0.000*
Neutrophils & Lymphocytes	0.968	21.757	0.000*
CD19+TLR-2+ [%] & CD4+TLR-4+	0.970	22.555	0.000*
White blood cells & Neutrophils	0.976	25.188	0.000*
sTLR-2 & sTLR-9 [ng/mL]	0.986	33.281	0.000*
CD8+TLR-9+ [%] & CD8+TLR-3+	0.987	34.854	0.000*
CD19+TLR-9+ [%] & CD19+TLR-3+	0.996	60.945	0.000*
CD8+TLR-2+ [%] & CD4+TLR-4+	0.997	71.288	0.000*
CD4+TLR-4+ & CD8+TLR-4+	0.997	71.288	0.000*
CD4+TLR-2+ [%] & CD8+TLR-2+ [%]	0.998	87.918	0.000*
CD4+TLR-2+ [%] & CD8+TLR-4+	0.998	87.918	0.000*
CD4+TLR-2+ [%] & CD4+TLR-4+	0.999	131.990	0.000*

*statistically significant results

Supplementary Material Table S8 Tabulated summary of Spearman's rank correlations for male patients diagnosed with GC.

Para zmiennych	R	t(N-2)	p
CD3+CD8+ [%] & CD8+TLR-2+ [%]	-0.335	-2.194	0.034*
CD3+CD8+ [%] & CD8+TLR-4+	-0.332	-2.170	0.036*
CD3+CD8+ [%] & CD4+TLR-2+ [%]	-0.319	-2.074	0.045*
CD3+CD8+ [%] & CD4+TLR-4+	-0.319	-2.074	0.045*
Age & CD8+TLR-9+ [%]	0.324	2.110	0.042*
CD3+CD4+ [%] & CD4+TLR-2+ [%]	0.330	2.158	0.037*
CD3+CD4+ [%] & CD4+TLR-4+	0.330	2.158	0.037*
CD3+CD4+ [%] & CD3+CD8+ [%]	0.331	2.163	0.037*
Lymphocytes & CD3+CD4+ [%]	0.335	2.193	0.034*
Age & sTLR-3	0.336	2.201	0.034*
CD3+ [%] & ratio CD3+CD4+/T CD3+CD8+	0.353	2.326	0.025*
Age & CD3+CD4+ [%]	0.354	2.331	0.025*
CD3+CD4+ [%] & Age	0.354	2.331	0.025*
CD3+CD4+ [%] & CD4+TLR-9+ [%]	0.368	2.438	0.020*
CD3+CD4+ [%] & CD19+TLR-3+	0.370	2.457	0.019*
CD3+CD4+ [%] & CD4+TLR-3+	0.378	2.520	0.016*
CD3+ [%] & CD8+TLR-3+	0.385	2.569	0.014*
CD3+ [%] & CD19+TLR-2+ [%]	0.385	2.569	0.014*
sTLR-2 & sTLR-3	0.387	2.587	0.014*
sTLR-3 & sTLR-9 [ng/mL]	0.390	2.612	0.013*
CD3+ [%] & CD19+TLR-4+	0.392	2.623	0.012*
CD3+ [%] & CD8+TLR-9+ [%]	0.400	2.691	0.011*
CD3+CD4+ [%] & CD19+TLR-9+ [%]	0.401	2.695	0.010*

CD3+CD4+ [%] & sTLR-2	0.408	2.757	0.009*
CD3+CD4+ [%] & CD19+TLR-2+ [%]	0.411	2.779	0.008*
CD3+CD4+ [%] & sTLR-3	0.412	2.788	0.008*
Lymphocytes & CD3+ [%]	0.414	2.803	0.008*
CD3+ [%] & CD19+TLR-3+	0.419	2.845	0.007*
Monocytes & CD3+CD4+ [%]	0.422	2.866	0.007*
CD3+CD4+ [%] & CD19+TLR-4+	0.422	2.872	0.007*
Stage& CD19+TLR-9+ [%]	0.431	2.741	0.010*
CD3+ [%] & CD19+TLR-9+ [%]	0.433	2.957	0.005*
Stage& CD19+TLR-3+	0.443	2.839	0.008*
Stage& CD4+TLR-9+ [%]	0.446	2.862	0.007
CD3+CD4+ [%] & sTLR-9 [ng/mL]	0.447	3.083	0.00*4
Stage& sTLR-9 [ng/mL]	0.455	2.933	0.006
Stage& CD4+TLR-3+	0.467	3.031	0.005
Stage& sTLR-2	0.472	3.074	0.004
CD3+ [%] & sTLR-2	0.477	3.349	0.002
CD3+CD4+ [%] & CD8+TLR-3+	0.487	3.439	0.001
Stage& CD19+TLR-2+ [%]	0.497	3.288	0.002
Stage& CD19+TLR-4+	0.497	3.288	0.002
White blood cells & CD3+ [%]	0.500	3.563	0.001
Neutrophils & CD3+CD4+ [%]	0.504	3.596	0.001
Neutrophils & CD3+ [%]	0.506	3.618	0.001
CD3+CD8+ [%] & sTLR-3	0.507	3.622	0.001
CD3+ [%] & sTLR-9 [ng/mL]	0.513	3.683	0.001*
CD3+CD4+ [%] & sTLR-4	0.515	3.707	0.001*
CD3+ [%] & sTLR-3	0.516	3.715	0.001*
Stage& sTLR-4	0.517	3.466	0.001*
Stage& CD8+TLR-9+ [%]	0.519	3.490	0.001*
ratio CD3+CD4+/T CD3+CD8+ & sTLR-4	0.521	3.762	0.001*
Stage& CD8+TLR-3+	0.521	3.506	0.001*
Stage& CD8+TLR-2+ [%]	0.533	3.618	0.001*
Stage& CD8+TLR-4+	0.533	3.618	0.001*
Monocytes & CD3+ [%]	0.540	3.953	0.000*
CD3+CD4+ [%] & CD8+TLR-9+ [%]	0.540	3.958	0.000*
Stage& CD4+TLR-2+ [%]	0.540	3.690	0.001*
Stage& CD4+TLR-4+	0.540	3.690	0.001*
Lymphocytes & CD8+TLR-4+	0.542	3.977	0.000*
White blood cells & CD3+CD4+ [%]	0.543	3.984	0.000*
CD3+ [%] & CD3+CD8+ [%]	0.543	3.988	0.000*
Lymphocytes & CD8+TLR-2+ [%]	0.543	3.989	0.000*
Lymphocytes & CD4+TLR-2+ [%]	0.544	3.996	0.000*
Lymphocytes & CD4+TLR-4+	0.544	3.996	0.000*
Lymphocytes & CD4+TLR-3+	0.558	4.140	0.000*
ratio CD3+CD4+/T CD3+CD8+ & CD8+TLR-2+ [%]	0.572	4.294	0.000*
ratio CD3+CD4+/T CD3+CD8+ & CD8+TLR-4+	0.572	4.303	0.000*
Lymphocytes & CD4+TLR-9+ [%]	0.577	4.353	0.000*
ratio CD3+CD4+/T CD3+CD8+ & CD4+TLR-2+ [%]	0.578	4.363	0.000*
ratio CD3+CD4+/T CD3+CD8+ & CD4+TLR-4+	0.578	4.363	0.000*
ratio CD3+CD4+/T CD3+CD8+ & sTLR-2	0.582	4.407	0.000*
Lymphocytes & sTLR-4	0.582	4.410	0.000*
ratio CD3+CD4+/T CD3+CD8+ & CD4+TLR-3+	0.587	4.474	0.000*
Lymphocytes & CD8+TLR-3+	0.588	4.486	0.000*
ratio CD3+CD4+/T CD3+CD8+ & CD19+TLR-3+	0.589	4.491	0.000*
ratio CD3+CD4+/T CD3+CD8+ & sTLR-9 [ng/mL]	0.597	4.593	0.000*
Lymphocytes & CD19+TLR-2+ [%]	0.600	4.623	0.000*
CD3+ [%] & sTLR-4	0.602	4.653	0.000*
Lymphocytes & CD19+TLR-4+	0.605	4.685	0.000*
Monocytes & ratio CD3+CD4+/T CD3+CD8+	0.620	4.877	0.000*
CD4+TLR-9+ [%] & sTLR-4	0.626	4.943	0.000*
ratio CD3+CD4+/T CD3+CD8+ & CD19+TLR-9+ [%]	0.627	4.958	0.000*
Lymphocytes & CD8+TLR-9+ [%]	0.628	4.969	0.000*
ratio CD3+CD4+/T CD3+CD8+ & CD4+TLR-9+ [%]	0.633	5.046	0.000*
sTLR-4 & sTLR-3	0.633	5.047	0.000*
CD3+ [%] & CD3+CD4+ [%]	0.634	5.057	0.000*
Lymphocytes & ratio CD3+CD4+/T CD3+CD8+	0.635	5.061	0.000*
ratio CD3+CD4+/T CD3+CD8+ & CD19+TLR-2+ [%]	0.644	5.193	0.000*

Lymphocytes & sTLR-2	0.650	5.271	0.000*
Lymphocytes & CD19+TLR-3+	0.652	5.299	0.000*
ratio CD3+CD4+/T CD3+CD8+ & CD19+TLR-4+	0.656	5.354	0.000*
Lymphocytes & sTLR-9 [ng/mL]	0.666	5.499	0.000*
CD3+CD4+ [%] & ratio CD3+CD4+/T CD3+CD8+	0.666	5.506	0.000*
ratio CD3+CD4+/T CD3+CD8+ & CD8+TLR-3+	0.669	5.556	0.000*
Monocytes & sTLR-4	0.670	5.556	0.000*
CD4+TLR-3+ & sTLR-4	0.682	5.752	0.000*
Lymphocytes & CD19+TLR-9+ [%]	0.683	5.771	0.000*
Monocytes & CD8+TLR-4+	0.685	5.790	0.000*
Monocytes & CD8+TLR-2+ [%]	0.686	5.813	0.000*
CD8+TLR-9+ [%] & sTLR-4	0.686	5.819	0.000*
Monocytes & CD4+TLR-2+ [%]	0.689	5.867	0.000*
Monocytes & CD4+TLR-4+	0.689	5.867	0.000*
CD8+TLR-3+ & sTLR-4	0.704	6.113	0.000*
White blood cells & sTLR-4	0.715	6.297	0.000*
CD8+TLR-2+ [%] & sTLR-4	0.721	6.406	0.000*
CD8+TLR-4+ & sTLR-4	0.721	6.416	0.000*
Neutrophils & sTLR-4	0.728	6.548	0.000*
ratio CD3+CD4+/T CD3+CD8+ & CD8+TLR-9+ [%]	0.732	6.617	0.000*
CD4+TLR-2+ [%] & sTLR-4	0.735	6.675	0.000*
CD4+TLR-4+ & sTLR-4	0.735	6.675	0.000*
Monocytes & CD4+TLR-9+ [%]	0.739	6.761	0.000*
CD19+TLR-9+ [%] & sTLR-4	0.744	6.859	0.000*
Monocytes & CD4+TLR-3+	0.745	6.877	0.000*
CD19+TLR-4+ & sTLR-4	0.745	6.881	0.000*
CD19+TLR-2+ [%] & sTLR-4	0.747	6.928	0.000*
Neutrophils & Lymphocytes [10^3/mm^3]	0.748	6.957	0.000*
White blood cells & Lymphocytes [10^3/mm^3]	0.756	7.128	0.000*
CD19+TLR-3+ & sTLR-4	0.757	7.148	0.000*
Neutrophils & ratio CD3+CD4+/T CD3+CD8+	0.757	7.150	0.000*
CD4+TLR-9+ [%] & CD19+TLR-9+ [%]	0.759	7.178	0.000*
CD4+TLR-9+ [%] & CD19+TLR-3+	0.762	7.246	0.000*

Monocytes & Lymphocytes [10^3/mm^3]	0.768	7.392	0.000*
Monocytes & CD8+TLR-3+	0.770	7.438	0.000*
CD8+TLR-9+ [%] & sTLR-9 [ng/mL]	0.775	7.554	0.000*
Monocytes & sTLR-2	0.778	7.635	0.000*
CD8+TLR-9+ [%] & sTLR-2	0.779	7.651	0.000*
Monocytes & CD8+TLR-9+ [%]	0.785	7.819	0.000*
Monocytes & sTLR-9 [ng/mL]	0.787	7.873	0.000*
Monocytes & CD19+TLR-3+	0.790	7.954	0.000*
CD19+TLR-9+ [%] & CD4+TLR-3+	0.792	8.000	0.000*
Neutrophils & CD8+TLR-4+	0.795	8.082	0.000*
CD8+TLR-3+ & sTLR-9 [ng/mL]	0.795	8.083	0.000*
Neutrophils & CD8+TLR-2+ [%]	0.796	8.113	0.000*
Neutrophils & CD4+TLR-2+ [%]	0.798	8.166	0.000*
Neutrophils & CD4+TLR-4+	0.798	8.166	0.000*
White blood cells & CD8+TLR-3+	0.799	8.182	0.000*
White blood cells & sTLR-2	0.799	8.200	0.000*
Neutrophils & CD4+TLR-9+ [%]	0.801	8.258	0.000*
Neutrophils & CD4+TLR-3+	0.802	8.289	0.000*
CD4+TLR-9+ [%] & sTLR-9 [ng/mL]	0.803	8.300	0.000*
CD4+TLR-3+ & CD19+TLR-3+	0.804	8.330	0.000*
White blood cells & CD19+TLR-3+	0.806	8.381	0.000*
CD4+TLR-9+ [%] & sTLR-2	0.807	8.422	0.000*
CD8+TLR-4+ & sTLR-9 [ng/mL]	0.809	8.480	0.000*
CD8+TLR-9+ [%] & CD19+TLR-3+	0.810	8.528	0.000*
White blood cells & sTLR-9 [ng/mL]	0.811	8.530	0.000*
CD8+TLR-2+ [%] & sTLR-9 [ng/mL]	0.811	8.531	0.000*
White blood cells & CD4+TLR-3+	0.811	8.533	0.000*
CD8+TLR-2+ [%] & CD8+TLR-9+ [%]	0.811	8.554	0.000*
White blood cells & CD8+TLR-4+	0.811	8.559	0.000*
White blood cells & CD8+TLR-2+ [%]	0.812	8.570	0.000*
White blood cells & ratio CD3+CD4+/T CD3+CD8+	0.812	8.579	0.000*
Monocytes & CD19+TLR-2+ [%]	0.812	8.588	0.000*
CD4+TLR-2+ [%] & CD8+TLR-9+ [%]	0.813	8.592	0.000*

CD8+TLR-9+ [%] & CD4+TLR-4+	0.813	8.592	0.000*
CD8+TLR-3+ & sTLR-2	0.814	8.630	0.000*
White blood cells & CD4+TLR-2+ [%]	0.814	8.644	0.000*
White blood cells & CD4+TLR-4+	0.814	8.644	0.000*
CD4+TLR-2+ [%] & sTLR-9 [ng/mL]	0.815	8.663	0.000*
sTLR-9 [ng/mL] & CD4+TLR-4+	0.815	8.663	0.000*
CD4+TLR-9+ [%] & CD8+TLR-9+ [%]	0.815	8.670	0.000*
Monocytes & CD19+TLR-9+ [%]	0.816	8.709	0.000*
Monocytes & CD19+TLR-4+	0.818	8.751	0.000*
White blood cells & CD4+TLR-9+ [%]	0.820	8.831	0.000*
White blood cells & CD8+TLR-9+ [%]	0.822	8.909	0.000*
White blood cells & CD19+TLR-9+ [%]	0.826	9.034	0.000*
CD8+TLR-9+ [%] & CD19+TLR-9+ [%]	0.826	9.047	0.000*
White blood cells & Monocytes [10^3/mm^3]	0.827	9.059	0.000*
CD8+TLR-9+ [%] & CD4+TLR-3+	0.827	9.072	0.000*
Neutrophils & CD19+TLR-3+	0.833	9.265	0.000*
CD4+TLR-9+ [%] & CD8+TLR-3+	0.833	9.272	0.000*
Neutrophils & sTLR-2	0.834	9.309	0.000*
CD4+TLR-3+ & sTLR-9 [ng/mL]	0.838	9.463	0.000*
Neutrophils & CD8+TLR-3+	0.839	9.517	0.000*
CD19+TLR-9+ [%] & CD8+TLR-4+	0.840	9.555	0.000*
CD8+TLR-2+ [%] & CD19+TLR-9+ [%]	0.841	9.584	0.000*
CD4+TLR-2+ [%] & CD19+TLR-9+ [%]	0.842	9.606	0.000*
CD19+TLR-9+ [%] & CD4+TLR-4+	0.842	9.606	0.000*
Neutrophils & sTLR-9 [ng/mL]	0.842	9.619	0.000*
CD19+TLR-9+ [%] & sTLR-9 [ng/mL]	0.843	9.663	0.000*
CD8+TLR-4+ & sTLR-2	0.845	9.744	0.000*
CD4+TLR-3+ & sTLR-2	0.846	9.768	0.000*
CD8+TLR-2+ [%] & sTLR-2	0.847	9.835	0.000*
CD19+TLR-9+ [%] & CD8+TLR-3+	0.847	9.841	0.000*
CD4+TLR-2+ [%] & sTLR-2	0.850	9.941	0.000*
sTLR-2 & CD4+TLR-4+	0.850	9.941	0.000*
CD19+TLR-9+ [%] & sTLR-2	0.850	9.967	0.000*

Neutrophils & CD19+TLR-9+ [%]	0.852	10.051	0.000*
CD19+TLR-2+ [%] & CD8+TLR-9+ [%]	0.853	10.055	0.000*
CD8+TLR-3+ & CD19+TLR-3+	0.854	10.124	0.000*
CD19+TLR-3+ & sTLR-9 [ng/mL]	0.856	10.203	0.000*
Neutrophils & CD8+TLR-9+ [%]	0.857	10.233	0.000*
CD4+TLR-9+ [%] & CD8+TLR-4+	0.857	10.237	0.000*
CD8+TLR-2+ [%] & CD4+TLR-9+ [%]	0.858	10.313	0.000*
CD19+TLR-4+ & sTLR-9 [ng/mL]	0.859	10.334	0.000*
CD19+TLR-2+ [%] & sTLR-9 [ng/mL]	0.861	10.439	0.000*
CD4+TLR-2+ [%] & CD4+TLR-9+ [%]	0.861	10.439	0.000*
CD4+TLR-9+ [%] & CD4+TLR-4+	0.861	10.439	0.000*
CD8+TLR-9+ [%] & CD19+TLR-4+	0.862	10.470	0.000*
CD4+TLR-3+ & CD8+TLR-3+	0.866	10.669	0.000*
CD19+TLR-4+ & sTLR-2	0.871	10.906	0.000*
CD19+TLR-3+ & sTLR-2	0.871	10.930	0.000*
CD8+TLR-4+ & CD19+TLR-3+	0.874	11.081	0.000*
CD8+TLR-2+ [%] & CD19+TLR-3+	0.874	11.112	0.000*
CD4+TLR-2+ [%] & CD19+TLR-3+	0.875	11.122	0.000*
CD19+TLR-3+ & CD4+TLR-4+	0.875	11.122	0.000*
CD19+TLR-2+ [%] & sTLR-2	0.876	11.206	0.000*
CD8+TLR-4+ & CD8+TLR-3+	0.882	11.563	0.000*
CD19+TLR-9+ [%] & CD19+TLR-4+	0.883	11.571	0.000*
CD8+TLR-2+ [%] & CD8+TLR-3+	0.883	11.596	0.000*
CD4+TLR-2+ [%] & CD8+TLR-3+	0.883	11.607	0.000*
CD8+TLR-3+ & CD4+TLR-4+	0.883	11.607	0.000*
CD19+TLR-2+ [%] & CD19+TLR-9+ [%]	0.883	11.626	0.000*
White blood cells & CD19+TLR-2+ [%]	0.885	11.720	0.000*
Neutrophils & CD19+TLR-2+ [%]	0.889	11.997	0.000*
CD19+TLR-4+ & CD19+TLR-3+	0.893	12.211	0.000*
White blood cells & CD19+TLR-4+	0.893	12.252	0.000*
CD19+TLR-2+ [%] & CD4+TLR-9+ [%]	0.894	12.306	0.000*
CD19+TLR-2+ [%] & CD8+TLR-3+	0.895	12.358	0.000*
CD4+TLR-9+ [%] & CD19+TLR-4+	0.896	12.424	0.000*

Neutrophils & CD19+TLR-4+	0.896	12.427	0.000*
CD19+TLR-4+ & CD8+TLR-3+	0.896	12.444	0.000*
CD8+TLR-4+ & CD4+TLR-3+	0.897	12.517	0.000*
CD19+TLR-2+ [%] & CD19+TLR-3+	0.898	12.564	0.000*
CD8+TLR-2+ [%] & CD4+TLR-3+	0.898	12.612	0.000*
CD4+TLR-2+ [%] & CD4+TLR-3+	0.902	12.842	0.000*
CD4+TLR-3+ & CD4+TLR-4+	0.902	12.842	0.000*
sTLR-4 & sTLR-9 [ng/mL]	0.916	14.099	0.000*
sTLR-2 & sTLR-4	0.917	14.181	0.000*
CD19+TLR-4+ & CD4+TLR-3+	0.925	14.993	0.000*
CD19+TLR-2+ [%] & CD4+TLR-3+	0.925	15.036	0.000*
Neutrophils & Monocytes [10^3/mm^3]	0.928	15.333	0.000*
White blood cells & Neutrophils [10^3/mm^3]	0.935	16.274	0.000*
CD8+TLR-4+ & CD19+TLR-4+	0.954	19.598	0.000*
CD8+TLR-2+ [%] & CD19+TLR-4+	0.954	19.641	0.000*
CD4+TLR-2+ [%] & CD19+TLR-4+	0.957	20.389	0.000*
CD19+TLR-4+ & CD4+TLR-4+	0.957	20.389	0.000*
CD19+TLR-2+ [%] & CD8+TLR-4+	0.962	21.660	0.000*
CD8+TLR-2+ [%] & CD19+TLR-2+ [%]	0.962	21.717	0.000*
CD4+TLR-2+ [%] & CD19+TLR-2+ [%]	0.965	22.524	0.000*
CD19+TLR-2+ [%] & CD4+TLR-4+	0.965	22.524	0.000*
CD8+TLR-9+ [%] & CD8+TLR-3+	0.975	27.231	0.000*
CD4+TLR-9+ [%] & CD4+TLR-3+	0.982	32.427	0.000*
sTLR-2 & sTLR-9 [ng/mL]	0.993	50.670	0.000*
CD4+TLR-2+ [%] & CD8+TLR-2+ [%]	0.998	91.697	0.000*
CD8+TLR-2+ [%] & CD4+TLR-4+	0.998	91.697	0.000*
CD4+TLR-2+ [%] & CD8+TLR-4+	0.998	105.927	0.000*
CD8+TLR-4+ & CD4+TLR-4+	0.998	105.927	0.000*
CD19+TLR-2+ [%] & CD19+TLR-4+	0.999	129.783	0.000*
CD8+TLR-2+ [%] & CD8+TLR-4+	1.000	318.140	0.000*

Supplementary Material Table S9 Tabulated summary of Spearman's rank correlations for male patients diagnosed with GC.

Para zmiennych	R	t(N-2)	p
CD3+CD8+ [%] & CD8+TLR-2+ [%]	-0.535	-3.228	0.003*
CD4+TLR-2+ [%] & CD3+CD8+ [%]	-0.527	-3.163	0.004*
CD3+CD8+ [%] & CD4+TLR-4+	-0.520	-3.104	0.005*
CD3+CD8+ [%] & CD19+TLR-2+ [%]	-0.484	-2.822	0.009*
CD3+CD8+ [%] & CD19+TLR-4+	-0.460	-2.641	0.014*
White blood cells & CD19+ [%]	0.379	2.091	0.046*
Neutrophils & CD3+ [%]	0.383	2.112	0.044*
Monocytes & CD19+ [%]	0.383	2.113	0.044*
Lymphocytes & CD19+ [%]	0.385	2.129	0.043*
CD3+ [%] & sTLR-3	0.386	2.136	0.042
CD3+ [%] & CD19+TLR-3+	0.392	2.175	0.039
CD3+CD4+ [%] & CD4+TLR-3+	0.395	2.194	0.037
CD3+ [%] & CD19+ [%]	0.399	2.220	0.035
CD19+ [%] & sTLR-2	0.400	2.225	0.035
CD8+TLR-9+ [%] & Age	0.418	2.343	0.027*
Monocytes & Age	0.427	2.409	0.023*
CD3+ [%] & sTLR-9 [ng/mL]	0.434	2.459	0.021*
CD3+ [%] & CD19+TLR-9+ [%]	0.435	2.464	0.021*
CD3+ [%] & ratio CD3+CD4+/T CD3+CD8+	0.438	2.484	0.020*
CD19+ [%] & sTLR-9 [ng/mL]	0.451	2.575	0.016*
Monocytes & CD8+TLR-2+ [%]	0.451	2.577	0.016*
Monocytes & CD8+TLR-4+	0.451	2.577	0.016*
Stage & sTLR-4	0.455	2.343	0.029*
CD3+CD4+ [%] & sTLR-4	0.458	2.630	0.014*
CD3+ [%] & sTLR-4	0.463	2.663	0.013*
Monocytes & CD4+TLR-2+ [%]	0.463	2.664	0.013*
Monocytes & CD4+TLR-4+	0.472	2.733	0.011*
CD3+ [%] & CD3+CD8+ [%]	0.478	2.776	0.010*
Lymphocytes & CD8+TLR-2+ [%]	0.486	2.833	0.009*

Lymphocytes & CD8+TLR-4+	0.486	2.833	0.009*
CD19+ [%] & CD3+CD4+ [%]	0.486	2.838	0.009*
CD19+ [%] & ratio CD3+CD4+/T CD3+CD8+	0.487	2.844	0.009*
CD3+CD4+ [%] & sTLR-2	0.494	2.899	0.008*
Lymphocytes & CD4+TLR-2+ [%]	0.500	2.942	0.007*
Monocytes & CD19+TLR-2+ [%]	0.502	2.960	0.006*
Lymphocytes & CD4+TLR-4+	0.510	3.020	0.006*
Monocytes & CD19+TLR-4+	0.517	3.078	0.005*
CD3+CD4+ [%] & CD8+TLR-2+ [%]	0.522	3.124	0.004*
CD3+CD4+ [%] & CD8+TLR-4+	0.522	3.124	0.004*
CD4+TLR-9+ [%] & sTLR-4	0.524	3.139	0.004*
Lymphocytes & CD3+ [%]	0.526	3.154	0.004*
sTLR-2 & sTLR-3	0.535	3.226	0.003*
White blood cells & Monocytes [10^3/mm^3]	0.536	3.237	0.003*
CD3+CD4+ [%] & CD4+TLR-2+ [%]	0.537	3.248	0.003*
Lymphocytes & CD19+TLR-2+ [%]	0.538	3.256	0.003*
CD3+CD4+ [%] & sTLR-9 [ng/mL]	0.541	3.281	0.003*
CD19+ [%] & CD19+TLR-3+	0.545	3.313	0.003*
Lymphocytes & CD3+CD4+ [%]	0.545	3.316	0.003*
CD3+CD4+ [%] & CD4+TLR-4+	0.547	3.333	0.003*
Neutrophils & Monocytes [10^3/mm^3]	0.547	3.335	0.003*
Lymphocytes & CD19+TLR-4+	0.553	3.387	0.002*
CD4+TLR-3+ & sTLR-4	0.556	3.407	0.002*
Monocytes & CD3+CD4+ [%]	0.561	3.453	0.002*
Stage & CD19+TLR-3+	0.563	3.122	0.005*
Monocytes & CD3+ [%]	0.572	3.553	0.001*
CD3+CD4+ [%] & CD19+TLR-2+ [%]	0.573	3.566	0.001*
sTLR-3 & sTLR-9 [ng/mL]	0.574	3.570	0.001*
CD3+CD4+ [%] & CD8+TLR-9+ [%]	0.574	3.571	0.001*
Stage & CD19+TLR-9+ [%]	0.575	3.220	0.004*
CD19+ [%] & CD19+TLR-9+ [%]	0.577	3.603	0.001*
CD8+TLR-9+ [%] & sTLR-4	0.586	3.689	0.001*
CD4+TLR-9+ [%] & sTLR-9 [ng/mL]	0.588	3.705	0.001*

CD3+CD4+ [%] & CD8+TLR-3+	0.588	3.706	0.001*
Lymphocytes & CD19+TLR-3+	0.589	3.717	0.001*
Monocytes & CD4+TLR-9+ [%]	0.591	3.736	0.001*
CD19+ [%] & CD8+TLR-3+	0.594	3.762	0.001*
CD8+TLR-3+ & sTLR-4	0.599	3.817	0.001*
CD4+TLR-9+ [%] & sTLR-2	0.601	3.839	0.001*
Neutrophils & CD3+CD4+ [%]	0.605	3.871	0.001*
Monocytes & sTLR-3	0.605	3.872	0.001*
CD19+TLR-3+ & sTLR-4	0.605	3.875	0.001*
White blood cells & sTLR-4	0.607	3.890	0.001*
Monocytes & CD4+TLR-3+	0.609	3.914	0.001*
CD19+TLR-9+ [%] & sTLR-4	0.611	3.935	0.001*
CD4+TLR-3+ & sTLR-9 [ng/mL]	0.614	3.969	0.001*
Lymphocytes & sTLR-3	0.617	3.993	0.000*
ratio CD3+CD4+/T CD3+CD8+ & CD4+TLR-9+ [%]	0.618	4.009	0.000*
Monocytes & ratio CD3+CD4+/T CD3+CD8+	0.624	4.069	0.000*
Lymphocytes & CD4+TLR-9+ [%]	0.628	4.120	0.000*
Monocytes & CD19+TLR-3+	0.630	4.133	0.000*
CD19+ [%] & CD8+TLR-9+ [%]	0.631	4.144	0.000*
Lymphocytes & CD19+TLR-9+ [%]	0.631	4.148	0.000*
Neutrophils & CD8+TLR-9+ [%]	0.632	4.154	0.000*
White blood cells & Lymphocytes [10^3/mm^3]	0.634	4.178	0.000*
CD4+TLR-3+ & sTLR-2	0.634	4.180	0.000*
CD8+TLR-9+ [%] & sTLR-9 [ng/mL]	0.636	4.202	0.000*
Neutrophils & CD8+TLR-3+	0.641	4.264	0.000*
Monocytes & CD8+TLR-9+ [%]	0.642	4.270	0.000*
ratio CD3+CD4+/T CD3+CD8+ & CD4+TLR-3+	0.642	4.271	0.000*
CD8+TLR-2+ [%] & sTLR-4	0.643	4.282	0.000*
sTLR-4 & CD8+TLR-4+	0.643	4.282	0.000*
CD8+TLR-2+ [%] & CD8+TLR-9+ [%]	0.644	4.290	0.000*
CD8+TLR-9+ [%] & CD8+TLR-4+	0.644	4.290	0.000*
Lymphocytes & ratio CD3+CD4+/T CD3+CD8+	0.645	4.303	0.000*
Lymphocytes & CD8+TLR-9+ [%]	0.646	4.315	0.000*

ratio CD3+CD4+/T CD3+CD8+ & sTLR-4	0.646	4.317	0.000*
CD4+TLR-2+ [%] & CD4+TLR-9+ [%]	0.647	4.324	0.000*
CD8+TLR-2+ [%] & CD4+TLR-9+ [%]	0.647	4.328	0.000*
CD4+TLR-9+ [%] & CD8+TLR-4+	0.647	4.328	0.000*
CD4+TLR-9+ [%] & CD4+TLR-4+	0.648	4.336	0.000*
CD8+TLR-9+ [%] & sTLR-2	0.650	4.358	0.000*
sTLR-9 [ng/mL] & CD8+TLR-3+	0.650	4.358	0.000*
Lymphocytes & CD4+TLR-3+	0.651	4.377	0.000*
CD4+TLR-2+ [%] & sTLR-4	0.652	4.386	0.000*
Lymphocytes & CD8+TLR-3+	0.654	4.411	0.000*
CD4+TLR-9+ [%] & CD19+TLR-4+	0.656	4.430	0.000*
CD3+ [%] & CD3+CD4+ [%]	0.656	4.435	0.000*
CD19+TLR-2+ [%] & CD8+TLR-9+ [%]	0.656	4.437	0.000*
Monocytes & CD8+TLR-3+	0.658	4.454	0.000*
CD4+TLR-4+ & sTLR-4	0.658	4.457	0.000*
CD4+TLR-2+ [%] & CD8+TLR-9+ [%]	0.658	4.459	0.000*
CD19+TLR-2+ [%] & CD4+TLR-9+ [%]	0.661	4.486	0.000*
CD8+TLR-9+ [%] & CD19+TLR-4+	0.662	4.502	0.000*
CD8+TLR-9+ [%] & CD4+TLR-4+	0.663	4.518	0.000*
Monocytes & sTLR-2	0.665	4.535	0.000*
CD8+TLR-2+ [%] & CD8+TLR-3+	0.667	4.559	0.000*
CD8+TLR-3+ & CD8+TLR-4+	0.667	4.559	0.000*
CD19+TLR-2+ [%] & sTLR-4	0.668	4.574	0.000*
CD19+TLR-4+ & sTLR-4	0.670	4.598	0.000*
CD8+TLR-3+ & sTLR-2	0.670	4.601	0.000*
White blood cells & sTLR-2	0.670	4.603	0.000*
CD19+TLR-2+ [%] & CD8+TLR-3+	0.676	4.676	0.000*
Neutrophils & CD4+TLR-9+ [%]	0.676	4.683	0.000*
Monocytes & CD19+TLR-9+ [%]	0.677	4.691	0.000*
CD4+TLR-2+ [%] & CD4+TLR-3+	0.678	4.698	0.000*
Stage & CD8+TLR-3+	0.679	4.234	0.000*
CD8+TLR-2+ [%] & CD4+TLR-3+	0.679	4.712	0.000*
CD4+TLR-3+ & CD8+TLR-4+	0.679	4.712	0.000*

Neutrophils & Lymphocytes [10^3/mm^3]	0.679	4.716	0.000*
CD19+TLR-4+ & CD8+TLR-3+	0.679	4.718	0.000*
CD4+TLR-2+ [%] & CD8+TLR-3+	0.680	4.728	0.000*
CD4+TLR-4+ & CD4+TLR-3+	0.681	4.740	0.000*
CD4+TLR-4+ & CD8+TLR-3+	0.685	4.792	0.000*
White blood cells & CD3+CD4+ [%]	0.685	4.794	0.000*
Neutrophils & sTLR-4	0.688	4.833	0.000*
CD19+TLR-4+ & CD4+TLR-3+	0.688	4.838	0.000*
White blood cells & CD8+TLR-9+ [%]	0.692	4.890	0.000*
CD19+TLR-2+ [%] & CD4+TLR-3+	0.694	4.908	0.000*
White blood cells & CD8+TLR-3+	0.699	4.988	0.000*
ratio CD3+CD4+/T CD3+CD8+ & sTLR-2	0.700	4.997	0.000*
Stage & CD8+TLR-9+ [%]	0.705	4.555	0.000*
Neutrophils & CD4+TLR-3+	0.705	5.069	0.000*
CD3+CD4+ [%] & CD19+TLR-3+	0.705	5.071	0.000*
White blood cells & sTLR-9 [ng/mL]	0.715	5.215	0.000*
CD19+TLR-3+ & sTLR-9 [ng/mL]	0.718	5.258	0.000*
Neutrophils & CD19+TLR-3+	0.719	5.273	0.000*
CD19+TLR-9+ [%] & sTLR-2	0.719	5.277	0.000*
CD4+TLR-9+ [%] & CD19+TLR-3+	0.720	5.289	0.000*
White blood cells & CD4+TLR-9+ [%]	0.722	5.314	0.000*
CD4+TLR-9+ [%] & CD19+TLR-9+ [%]	0.722	5.314	0.000*
Monocytes & sTLR-9 [ng/mL]	0.723	5.340	0.000*
CD19+TLR-9+ [%] & sTLR-9 [ng/mL]	0.726	5.382	0.000*
CD8+TLR-2+ [%] & CD19+TLR-9+ [%]	0.736	5.536	0.000*
CD19+TLR-9+ [%] & CD8+TLR-4+	0.736	5.536	0.000*
CD8+TLR-2+ [%] & sTLR-9 [ng/mL]	0.736	5.538	0.000*
sTLR-9 [ng/mL] & CD8+TLR-4+	0.736	5.538	0.000*
CD4+TLR-9+ [%] & CD8+TLR-9+ [%]	0.737	5.556	0.000*
White blood cells & CD4+TLR-3+	0.737	5.561	0.000*
ratio CD3+CD4+/T CD3+CD8+ & sTLR-9 [ng/mL]	0.737	5.568	0.000*
CD3+CD4+ [%] & CD19+TLR-9+ [%]	0.740	5.607	0.000*
CD4+TLR-2+ [%] & sTLR-9 [ng/mL]	0.741	5.620	0.000*

Lymphocytes & sTLR-2	0.741	5.630	0.000*
Neutrophils & CD19+TLR-9+ [%]	0.741	5.634	0.000*
CD19+TLR-3+ & sTLR-2	0.743	5.667	0.000*
CD19+TLR-9+ [%] & CD4+TLR-3+	0.744	5.674	0.000*
CD4+TLR-2+ [%] & CD19+TLR-9+ [%]	0.745	5.697	0.000*
CD4+TLR-3+ & CD19+TLR-3+	0.745	5.699	0.000*
CD4+TLR-4+ & sTLR-9 [ng/mL]	0.747	5.723	0.000*
sTLR-4 & sTLR-3	0.749	5.766	0.000*
CD8+TLR-9+ [%] & CD4+TLR-3+	0.750	5.785	0.000*
CD4+TLR-9+ [%] & CD8+TLR-3+	0.753	5.839	0.000*
CD19+TLR-9+ [%] & CD4+TLR-4+	0.753	5.841	0.000*
Monocytes & sTLR-4	0.757	5.904	0.000*
ratio CD3+CD4+/T CD3+CD8+ & CD8+TLR-9+ [%]	0.759	5.936	0.000*
Neutrophils & sTLR-2	0.760	5.969	0.000*
ratio CD3+CD4+/T CD3+CD8+ & CD8+TLR-3+	0.766	6.080	0.000*
CD19+TLR-2+ [%] & CD19+TLR-9+ [%]	0.767	6.096	0.000*
White blood cells & CD19+TLR-3+	0.769	6.141	0.000*
CD19+TLR-2+ [%] & sTLR-9 [ng/mL]	0.770	6.157	0.000*
CD4+TLR-3+ & CD8+TLR-3+	0.772	6.195	0.000*
CD19+TLR-4+ & sTLR-9 [ng/mL]	0.775	6.250	0.000*
CD19+TLR-9+ [%] & CD19+TLR-4+	0.777	6.303	0.000*
CD8+TLR-2+ [%] & CD19+TLR-3+	0.786	6.485	0.000*
CD19+TLR-3+ & CD8+TLR-4+	0.786	6.485	0.000*
CD19+TLR-4+ & sTLR-2	0.792	6.605	0.000*
ratio CD3+CD4+/T CD3+CD8+ & CD8+TLR-2+ [%]	0.792	6.608	0.000*
ratio CD3+CD4+/T CD3+CD8+ & CD8+TLR-4+	0.792	6.608	0.000*
White blood cells & CD19+TLR-9+ [%]	0.792	6.620	0.000*
CD4+TLR-2+ [%] & CD19+TLR-3+	0.793	6.642	0.000*
Lymphocytes & sTLR-9 [ng/mL]	0.794	6.667	0.000*
CD19+TLR-2+ [%] & CD19+TLR-3+	0.795	6.679	0.000*
CD19+TLR-4+ & CD19+TLR-3+	0.795	6.692	0.000*
CD4+TLR-4+ & CD19+TLR-3+	0.798	6.756	0.000*
Neutrophils & CD8+TLR-2+ [%]	0.801	6.819	0.000*
Neutrophils & CD8+TLR-4+	0.801	6.819	0.000*
CD19+TLR-2+ [%] & sTLR-2	0.803	6.865	0.000*
CD8+TLR-2+ [%] & sTLR-2	0.804	6.892	0.000*
sTLR-2 & CD8+TLR-4+	0.804	6.892	0.000*
CD4+TLR-2+ [%] & sTLR-2	0.804	6.892	0.000*
Neutrophils & sTLR-9 [ng/mL]	0.804	6.905	0.000*
Monocytes & Lymphocytes [10^3/mm^3]	0.805	6.910	0.000*
CD4+TLR-4+ & sTLR-2	0.806	6.946	0.000*
ratio CD3+CD4+/T CD3+CD8+ & CD4+TLR-2+ [%]	0.808	6.997	0.000*
Neutrophils & CD4+TLR-2+ [%]	0.812	7.105	0.000*
Lymphocytes & sTLR-4	0.816	7.204	0.000*
ratio CD3+CD4+/T CD3+CD8+ & CD4+TLR-4+	0.818	7.252	0.000*
Neutrophils & CD4+TLR-4+	0.822	7.353	0.000*
White blood cells & CD8+TLR-2+ [%]	0.829	7.561	0.000*
White blood cells & CD8+TLR-4+	0.829	7.561	0.000*
CD3+CD4+ [%] & ratio CD3+CD4+/T CD3+CD8+	0.837	7.809	0.000*
Neutrophils & ratio CD3+CD4+/T CD3+CD8+	0.838	7.843	0.000*
White blood cells & CD4+TLR-2+ [%]	0.843	8.004	0.000*
White blood cells & CD4+TLR-4+	0.852	8.284	0.000*
ratio CD3+CD4+/T CD3+CD8+ & CD19+TLR-2+ [%]	0.853	8.326	0.000*
ratio CD3+CD4+/T CD3+CD8+ & CD19+TLR-3+	0.867	8.884	0.000*
Neutrophils & CD19+TLR-2+ [%]	0.869	8.970	0.000*
ratio CD3+CD4+/T CD3+CD8+ & CD19+TLR-4+	0.873	9.117	0.000*
Neutrophils & CD19+TLR-4+	0.890	9.932	0.000*
ratio CD3+CD4+/T CD3+CD8+ & CD19+TLR-9+ [%]	0.893	10.143	0.000*
White blood cells & CD19+TLR-2+ [%]	0.898	10.413	0.000*
CD8+TLR-9+ [%] & CD19+TLR-3+	0.898	10.430	0.000*
CD8+TLR-9+ [%] & CD19+TLR-9+ [%]	0.901	10.581	0.000*
CD19+TLR-9+ [%] & CD8+TLR-3+	0.911	11.243	0.000*
CD8+TLR-3+ & CD19+TLR-3+	0.913	11.425	0.000*
White blood cells & ratio CD3+CD4+/T CD3+CD8+	0.919	11.891	0.000*
White blood cells & CD19+TLR-4+	0.920	11.995	0.000*
White blood cells & Neutrophils [10^3/mm^3]	0.921	12.065	0.000*

sTLR-2 & sTLR-4	0.927	12.577	0.000*
sTLR-4 & sTLR-9 [ng/mL]	0.946	14.948	0.000*
CD8+TLR-2+ [%] & CD19+TLR-4+	0.969	19.923	0.000*
CD19+TLR-4+ & CD8+TLR-4+	0.969	19.923	0.000*
sTLR-2 & sTLR-9 [ng/mL]	0.974	21.794	0.000*
CD4+TLR-2+ [%] & CD19+TLR-4+	0.974	22.028	0.000*
CD4+TLR-4+ & CD19+TLR-4+	0.979	24.592	0.000*
CD8+TLR-2+ [%] & CD19+TLR-2+ [%]	0.984	27.778	0.000*
CD19+TLR-2+ [%] & CD8+TLR-4+	0.984	27.778	0.000*
CD4+TLR-2+ [%] & CD19+TLR-2+ [%]	0.987	31.126	0.000*
CD19+TLR-9+ [%] & CD19+TLR-3+	0.987	31.643	0.000*
CD19+TLR-2+ [%] & CD4+TLR-4+	0.990	36.031	0.000*
CD4+TLR-9+ [%] & CD4+TLR-3+	0.992	39.868	0.000*
CD19+TLR-2+ [%] & CD19+TLR-4+	0.997	62.736	0.000*
CD8+TLR-2+ [%] & CD4+TLR-4+	0.997	68.735	0.000*
CD8+TLR-4+ & CD4+TLR-4+	0.997	68.735	0.000*
CD4+TLR-2+ [%] & CD8+TLR-2+ [%]	0.998	88.811	0.000*
CD4+TLR-2+ [%] & CD8+TLR-4+	0.998	88.811	0.000*
CD4+TLR-2+ [%] & CD4+TLR-4+	0.999	153.903	0.000*

* statistically significant results

Supplementary Material Table S10. Tabulated summary of ROC curves for type of GC.

	CD4+TLR-2+[%]			CD8+TLR-2+[%]			CD19+TLR-2+[%]		
	D vs. I	D vs. C	I vs. C	D vs. I	D vs. C	I vs. C	D vs. I	D vs. C	I vs. C
Area	0.745	0.961	0.879	0.743	0.895	0.689	0.741	0.883	0.668
Std. Error	0.070	0.022	0.048	0.070	0.042	0.077	0.071	0.047	0.074
95% confidence interval	0,6078 to 0,8821	0,9191 to 1,000	0,7845 to 0,9728	0,6046 to 0,8806	0,8135 to 0,9767	0,5373 to 0,8406	0,6022 to 0,8794	0,7902 to 0,9754	0,5235 to 0,8116
P value	0.001	<0,0001	<0,0001	0.002	<0,0001	0.019	0.002	<0,0001	0.035

	CD4+TLR-9+[%]			CD8+TLR-9+[%]			CD19+TLR-9+[%]		
	D vs. I	D vs. C	I vs. C	D vs. I	D vs. C	I vs. C	D vs. I	D vs. C	I vs. C
Area	0.756	1.000	1.000	0.782	1.000	1.000	0.774	1.000	1.000
Std. Error	0.065	0.000	0.000	0.063	0.000	0.000	0.065	0.000	0.000
95% confidence interval	0,6289 to 0,8836	1,000 to 1,000	1,000 to 1,000	0,6578 to 0,9058	1,000 to 1,000	1,000 to 1,000	0,6460 to 0,9021	1,000 to 1,000	1,000 to 1,000
P value	0.001	<0,0001	<0,0001	0.000	<0,0001	<0,0001	0.000	<0,0001	<0,0001

	CD4+TLR-4+[%]			CD8+TLR-4+[%]			CD19+TLR-4+[%]		
	D vs. I	D vs. C	I vs. C	D vs. I	D vs. C	I vs. C	D vs. I	D vs. C	I vs. C
Area	0.743	0.970	0.901	0.741	0.905	0.722	0.735	0.906	0.739
Std. Error	0.070	0.018	0.042	0.070	0.039	0.073	0.071	0.041	0.067

95% confidence interval	0,6059 to 0,8804	0,9337 to 1,000	0,8190 to 0,9837	0,6035 to 0,8793	0,8287 to 0,9810	0,5787 to 0,8654	0,5966 to 0,8731	0,8253 to 0,9871	0,6082 to 0,8704
P value	0.002	<0,0001	<0,0001	0.002	<0,0001	0.005	0.002	<0,0001	0.003

	CD4+TLR-3+[%]			CD8+TLR-3+[%]			CD19+TLR-3+[%]		
	D vs. I	D vs. C	I vs. C	D vs. I	D vs. C	I vs. C	D vs. I	D vs. C	I vs. C
Area	0.743	1.000	1.000	0.782	1.000	1.000	0.772	1.000	1.000
Std. Error	0.068	0.000	0.000	0.065	0.000	0.000	0.067	0.000	0.000
95% confidence interval	0,6104 to 0,8759	1,000 to 1,000	1,000 to 1,000	0,6549 to 0,9099	1,000 to 1,000	1,000 to 1,000	0,6405 to 0,9029	1,000 to 1,000	1,000 to 1,000
P value	0.002	<0,0001	<0,0001	0.000	<0,0001	<0,0001	0.000	<0,0001	<0,0001

	sTLR-2			sTLR-4			sTLR-3			sTLR-9		
	D vs. I	D vs. C	I vs. C	D vs. I	D vs. C	I vs. C	D vs. I	D vs. C	I vs. C	D vs. I	D vs. C	I vs. C
Area	0.791	1.000	1.000	0.763	1.000	1.000	0.570	1.000	1.000	0.791	1.000	1.000
Std. Error	0.068	0.000	0.000	0.069	0.000	0.000	0.077	0.000	0.000	0.068	0.000	0.000
95% confidence interval	0,6578 to 0,9237	1,000 to 1,000	1,000 to 1,000	0,6273 to 0,8983	1,000 to 1,000	1,000 to 1,000	0,4180 to 0,7211	1,000 to 1,000	1,000 to 1,000	0,6578 to 0,9237	1,000 to 1,000	1,000 to 1,000
P value	0.000	<0,0001	<0,0001	0.001	<0,0001	<0,0001	0.363	<0,0001	<0,0001	0.000	<0,0001	<0,0001

Supplementary Material Table S11. Tabulated summary of ROC curves for each stage.

CD4+TLR-2+[%]						CD8+TLR-2+[%]						CD19+TLR-2+[%]					
I vs. II	I vs. III	I vs. IV	II vs. III	II vs. IV	III vs. IV	I vs. II	I vs. III	I vs. IV	II vs. III	II vs. IV	III vs. IV	I vs. II	I vs. III	I vs. IV	II vs. III	II vs. IV	III vs. IV
0.5337	0.692	0.971	0.628	0.852	0.763	0.664	0.753	0.952	0.582	0.773	0.717	0.740	0.919	1.000	0.668	0.844	0.737
0.1099	0.093	0.034	0.097	0.080	0.094	0.104	0.086	0.045	0.099	0.100	0.102	0.092	0.046	0.000	0.095	0.079	0.102
0,3182 to 0,7491	0,5091 to 0,8755	0,9053 to 1,000	0,4391 to 0,8175	0,6949 to 1,000	0,5799 to 0,9464	0,4606 to 0,8663	0,5846 to 0,9215	0,8635 to 1,000	0,3877 to 0,7768	0,5771 to 0,9698	0,5166 to 0,9176	0,5592 to 0,9216	0,8284 to 1,000	1,000 to 1,000	0,4816 to 0,8540	0,6891 to 0,9984	0,5370 to 0,9366
0.7589	0.068	0.000	0.197	0.006	0.034	0.136	0.017	0.001	0.408	0.032	0.080	0.028	<0,0001	0.000	0.091	0.007	0.056
CD4+TLR-9+[%]						CD8+TLR-9+[%]						CD19+TLR-9+[%]					
I vs. II	I vs. III	I vs. IV	II vs. III	II vs. IV	III vs. IV	I vs. II	I vs. III	I vs. IV	II vs. III	II vs. IV	III vs. IV	I vs. II	I vs. III	I vs. IV	II vs. III	II vs. IV	III vs. IV
0.6587	0.785	0.981	0.618	0.836	0.796	0.505	0.729	0.981	0.671	0.852	0.704	0.548	0.781	0.942	0.707	0.836	0.684

0.1019	0.083	0.025	0.100	0.081	0.089	0.113	0.092	0.025	0.094	0.079	0.101	0.114	0.090	0.059	0.092	0.090	0.107
0,4589 to 0,8584	0,6224 to 0,9485	0,9324 to 1,000	0,4227 to 0,8141	0,6768 to 0,9951	0,6219 to 0,9702	0,2834 to 0,7262	0,5475 to 0,9099	0,9324 to 1,000	0,4875 to 0,8546	0,6971 to 1,000	0,5066 to 0,9013	0,3244 to 0,7718	0,6045 to 0,9582	0,8268 to 1,000	0,5269 to 0,8876	0,6600 to 1,000	0,4740 to 0,8944
0.1479	0.007	0.000	0.233	0.009	0.017	0.965	0.030	0.000	0.085	0.006	0.100	0.661	0.008	0.001	0.037	0.009	0.137
CD4+TLR-4+[%]						CD8+TLR-4+[%]						CD19+TLR-4+[%]					
I vs. II	I vs. III	I vs. IV	II vs. III	II vs. IV	III vs. IV	I vs. II	I vs. III	I vs. IV	II vs. III	II vs. IV	III vs. IV	I vs. II	I vs. III	I vs. IV	II vs. III	II vs. IV	III vs. IV
0.5769	0.737	0.990	0.638	0.852	0.757	0.563	0.729	0.990	0.645	0.859	0.757	0.534	0.692	0.971	0.628	0.852	0.763
0.1088	0.089	0.016	0.096	0.080	0.093	0.109	0.090	0.016	0.096	0.079	0.093	0.110	0.093	0.034	0.097	0.080	0.094
0,3638 to 0,7901	0,5620 to 0,9117	0,9600 to 1,000	0,4499 to 0,8264	0,6949 to 1,000	0,5735 to 0,9396	0,3487 to 0,7763	0,5515 to 0,9060	0,9600 to 1,000	0,4574 to 0,8321	0,7053 to 1,000	0,5735 to 0,9396	0,3182 to 0,7491	0,5091 to 0,8755	0,9053 to 1,000	0,4391 to 0,8175	0,6949 to 1,000	0,5799 to 0,9464
0.4829	0.025	0.000	0.164	0.006	0.038	0.569	0.030	0.000	0.145	0.005	0.038	0.759	0.068	0.000	0.197	0.006	0.034
CD4+TLR-3+[%]						CD8+TLR-3+[%]						CD19+TLR-3+[%]					
I vs. II	I vs. III	I vs. IV	II vs. III	II vs. IV	III vs. IV	I vs. II	I vs. III	I vs. IV	II vs. III	II vs. IV	III vs. IV	I vs. II	I vs. III	I vs. IV	II vs. III	II vs. IV	III vs. IV
0.6635	0.753	0.952	0.582	0.773	0.717	0.740	0.919	1.000	0.668	0.844	0.737	0.659	0.785	0.981	0.618	0.836	0.796
0.1035	0.086	0.045	0.099	0.100	0.102	0.092	0.046	0.000	0.095	0.079	0.102	0.102	0.083	0.025	0.100	0.081	0.089
0,4606 to 0,8663	0,5846 to 0,9215	0,8635 to 1,000	0,3877 to 0,7768	0,5771 to 0,9698	0,5166 to 0,9176	0,5592 to 0,9216	0,8284 to 1,000	1,000 to 1,000	0,4816 to 0,8540	0,6891 to 0,9984	0,5370 to 0,9366	0,4589 to 0,8584	0,6224 to 0,9485	0,9324 to 1,000	0,4227 to 0,8141	0,6768 to 0,9951	0,6219 to 0,9702
0.136	0.017	0.001	0.408	0.032	0.080	0.028	<0,0001	0.000	0.091	0.007	0.056	0.148	0.007	0.000	0.233	0.009	0.017

sTLR2						sTLR4						sTLR3						sTLR9					
I vs. II	I vs. III	I vs. IV	II vs. III	II vs. IV	III vs. IV	I vs. II	I vs. III	I vs. IV	II vs. III	II vs. IV	III vs. IV	I vs. II	I vs. III	I vs. IV	II vs. III	II vs. IV	III vs. IV	I vs. II	I vs. III	I vs. IV	II vs. III	II vs. IV	III vs. IV
0.5048	0.729	0.981	0.671	0.852	0.704	0.548	0.781	0.942	0.707	0.836	0.684	0.563	0.587	0.529	0.658	0.609	0.513	0.500	0.729	0.971	0.668	0.836	
0.113	0.092	0.025	0.094	0.079	0.101	0.114	0.090	0.059	0.092	0.090	0.107	0.111	0.103	0.149	0.095	0.128	0.125	0.113	0.092	0.033	0.094	0.082	
0,2834 to 0,7262	0,5475 to 0,9099	0,9324 to 1,000	0,4875 to 0,8546	0,6971 to 1,000	0,5066 to 0,9013	0,3244 to 0,7718	0,6045 to 0,9582	0,8268 to 1,000	0,5269 to 0,8876	0,6600 to 1,000	0,4740 to 0,8944	0,3451 to 0,7799	0,3848 to 0,7893	0,2373 to 0,8204	0,4719 to 0,8439	0,3577 to 0,8611	0,2682 to 0,7581	0,2795 to 0,7205	0,5475 to 0,9099	0,9061 to 1,000	0,4839 to 0,8516	0,6745 to 0,9974	
0.965	0.030	0.000	0.085	0.006	0.100	0.661	0.008	0.001	0.037	0.009	0.137	0.569	0.409	0.828	0.112	0.391	0.915	>0,9999	0.030	0.000	0.091	0.009	

Supplementary Material Table S12. Tabulated summary of ROC curves for gender differences and type of GC

	Male					Female		
	CD4+TLR-2+[%]	CD8+TLR-2+[%]	CD19+TLR-2+[%]			CD4+TLR-2+[%]	CD8+TLR-2+[%]	CD19+TLR-2+[%]
Area	0.776	0.772	0.769		Area	0.667	0.667	0.658
Std. Error	0.096	0.097	0.096		Std. Error	0.130	0.130	0.130
95% confidence interval	0,5868 to 0,9642	0,5814 to 0,9628	0,5804 to 0,9570		95% confidence interval	0,4120 to 0,9214	0,4120 to 0,9214	0,4035 to 0,9132
P value	0.0064	0.0071	0.0078		P value	0.1967	0.1967	0.22
	CD4+TLR-9+[%]	CD8+TLR-9+[%]	CD19+TLR-9+[%]			CD4+TLR-9+[%]	CD8+TLR-9+[%]	CD19+TLR-9+[%]
Area	0.762	0.796	0.772		Area	0.758	0.742	0.775
Std. Error	0.084	0.087	0.089		Std. Error	0.116	0.104	0.112
95% confidence interval	0,5964 to 0,9274	0,6256 to 0,9662	0,5969 to 0,9474		95% confidence interval	0,5316 to 0,9850	0,5381 to 0,9452	0,5562 to 0,9938
P value	0.0095	0.0034	0.0071		P value	0.0454	0.0612	0.0332
	CD4+TLR-4+[%]	CD8+TLR-4+[%]	CD19+TLR-4+[%]			CD4+TLR-4+[%]	CD8+TLR-4+[%]	CD19+TLR-4+[%]
Area	0.776	0.772	0.769		Area	0.667	0.667	0.658
Std. Error	0.096	0.097	0.096		Std. Error	0.130	0.130	0.130
95% confidence interval	0,5868 to 0,9642	0,5814 to 0,9628	0,5804 to 0,9570		95% confidence interval	0,4120 to 0,9214	0,4120 to 0,9214	0,4035 to 0,9132
P value	0.0064	0.0071	0.0078		P value	0.1967	0.1967	0.22
	CD4+TLR-3+[%]	CD8+TLR-3+[%]	CD19+TLR-3+[%]			CD4+TLR-3+[%]	CD8+TLR-3+[%]	CD19+TLR-3+[%]
Area	0.735	0.782	0.765		Area	0.758	0.758	0.775
Std. Error	0.096	0.096	0.095		Std. Error	0.116	0.102	0.112

95% confidence interval	0,5466 to 0,9228	0,5950 to 0,9696	0,5784 to 0,9522			95% confidence interval	0,5316 to 0,9850	0,5581 to 0,9586	0,5562 to 0,9938	
P value	0.0202	0.0052	0.0086			P value	0.0454	0.0454	0.0332	
	sTLR-2	sTLR-4	sTLR-3	sTLR-9			sTLR-2	sTLR-4	sTLR-3	sTLR-9
Area	0.796	0.731	0.605	0.605		Area	0.767	0.783	0.525	0.767
Std. Error	0.094	0.098	0.104	0.104		Std. Error	0.123	0.123	0.150	0.123
95% confidence interval	0,6121 to 0,9798	0,5399 to 0,9227	0,4012 to 0,8096	0,4012 to 0,8096		95% confidence interval	0,5266 to 1,000	0,5425 to 1,000	0,2302 to 0,8198	0,5266 to 1,000
P value	0.0034	0.022	0.2966	0.2966		P value	0.0389	0.0282	0.8465	0.0389
